# Screening and characterization of extracellular polysaccharides produced by *Leuconostoc kimchii* isolated from traditional fermented *pulque* beverage

**DOI:** 10.1186/2193-1801-3-583

**Published:** 2014-10-07

**Authors:** Ingrid Torres-Rodríguez, María Elena Rodríguez-Alegría, Alfonso Miranda-Molina, Martha Giles-Gómez, Rodrigo Conca Morales, Agustín López-Munguía, Francisco Bolívar, Adelfo Escalante

**Affiliations:** Departamento de Ingeniería Celular y Biocatálisis, Instituto de Biotecnología, Universidad Nacional Autónoma de México (UNAM), Av. Universidad 2001. Col. Chamilpa, Cuernavaca Morelos, 62210 México; Departamento de Biología, Facultad de Química, UNAM. Ciudad Universitaria, México D. F, Coyoacán, 04510 México

**Keywords:** *Pulque*, Lactic acid bacteria, *Leuconostoc kimchii*, Exopolysaccharides, Dextran, Levan

## Abstract

We report the screening and characterization of EPS produced by LAB identified as *Leuconostoc kimchii* isolated from *pulque*, a traditional Mexican fermented, non-distilled alcoholic beverage produced by the fermentation of the sap extracted from several (*Agave*) maguey species. EPS-producing LAB constitutes an abundant bacterial group relative to total LAB present in sap and during fermentation, however, only two EPS-producing colony phenotypes (EPSA and EPSB, respectively) were detected and isolated concluding that despite the high number of polymer-producing LAB their phenotypic diversity is low. Scanning electron microcopy analysis during EPS-producing conditions revealed that both types of EPS form a uniform porous structure surrounding the bacterial cells. The structural characterization of the soluble and cell-associated EPS fractions of each polymer by enzymatic and acid hydrolysis, as by 1D- and 2D-NMR, showed that polymers produced by the soluble and cell-associated fractions of EPSA strain are dextrans consisting of a linear backbone of linked α-(1→6) Glc*p* in the main chain with α-(1→2) and α-(1→3)-linked branches. The polymer produced by the soluble fraction of EPSB strain was identified as a class 1 dextran with a linear backbone containing consecutive α-(1→6)-linked D-glucopyranosyl units with few α-(1→3)-linked branches, whereas the cell-associated EPS is a polymer mixture consisting of a levan composed of linear chains of (2→6)-linked β-D-fructofuranosyl residues with β-(2→6) connections, and a class 1 dextran. According to our knowledge this is the first report of dextrans and a levan including their structural characterization produced by *L. kimchii* isolated from a traditional fermented source.

**Electronic supplementary material:**

The online version of this article (doi:10.1186/2193-1801-3-583) contains supplementary material, which is available to authorized users.

## Background

The analysis of the microbial diversity in traditional fermented beverages, cereal doughs and vegetables has revealed the presence of a remarkable diversity of LAB involved in the development of the characteristic sensorial properties of fermented foods (Giraffa 2004). A wide diversity of EPS and genes encoding biosynthetic enzymes from naturally occurring LAB in traditional fermented foods and beverages have been extensively studied for their role in the physicochemical and sensorial characteristics of final fermented products (viscosifying, stabilizing or water-binding agents). This has led to the discovery of a remarkable structural diversity of EPS produced by LAB, particularly of the genus *Leuconostoc* and *Weissella* (Chellapandian et al. 1998; Uzochukwu et al. 2001; Olivares-Illana et al. 2002; Uzochukwu et al. 2002; van Hijum et al. 2006; Eom et al. 2007; Van der Meulen et al. 2007; Bauer et al. 2009; Bounaix et al. 2009; Bounaix et al. 2010; Amari et al. 2012; Vasileva et al. 2012). EPS have received additional attention as valuable products because of their potential economic applications that include natural, safe-food additives or natural functional food ingredients for their properties as soluble fiber and prebiotics, and the possibility that they can replace or reduce the use of hydrocolloids (Giraffa 2004; Tieking et al. 2005; Vu et al. 2009; Leemhuis et al. 2013).

*Pulque* is a traditional Mexican, non-distilled alcoholic fermented beverage currently produced and consumed mainly in the Central Mexico Plateau. It is obtained from the fermentation of a fresh sap, known as *aguamiel*, which is extracted from several maguey (*Agave*) species, such as *Agave atrovensis* and *A. americana*. The production of this traditional beverage requires for the freshly collected *aguamiel* to be deposited in large open containers where previously fermented *pulque* acts as seed for a new batch. Fermentation time varies from a few hours, overnight, or even for several days. Fermented *pulque* is gradually retired from the container but always leaving a residual volume of the fermented beverage to start a new fermentation. The viscosity resultant from EPS synthesis and the alcohol content of the beverage are the main parameters used to determine the extent of fermentation as they define its distinctive sensorial properties (Escalante et al. 2008; Escalante et al. 2012).

The LAB *L. mesenteroides* has been traditionally considered one of the most important microorganisms during *pulque* fermentation as a result of its ability to synthesize EPS, primarily dextrans produced by a glucosyltransferase from sucrose present in *aguamiel* and *pulque* (Sanchez-Marroquin and Hope 1953; Chellapandian et al. 1998; Escalante et al. 2012). Although EPS production by *Leuconostoc* species isolated from *pulque* was first reported in 1953, no detailed information was available concerning the properties or structure of these polymers (Sanchez-Marroquin and Hope 1953). Nevertheless, the structure of an EPS produced by *L. mesenteroides* strain IBT-PQ isolated from *pulque*, revealed the presence of a soluble linear dextran with glucose molecules linked primarily by α-(1→6) bonds with branching from α-(1→3) bonds, in a 4:1 ratio, and was produced by a cell-associated dextransucrase displaying a similar biochemical behavior to that reported for this enzyme obtained from the industrial strain *L. mesenteroides* NRRL B512F (Chellapandian et al. 1998).

We have previously reported a great LAB diversity in *aguamiel* and *pulque* samples from different geographical origins, that is composed mainly of *Lactobacillus* and *Leuconostoc* species (Escalante et al. 2004). Among them *L. citreum* and *L. kimchii* were reported for the first time to be the most abundant LAB present in *aguamiel* and during the early stages of *pulque* fermentation (Escalante et al. 2008). This remarkable abundance of *L. citreum* and *L. kimchii* in *aguamiel* and *pulque*, suggests the presence of a possible diversity of EPS produced by these LAB during fermentation contributing to the final sensorial properties of the beverage. The aim of the present work was to screen and characterize the EPS diversity associated to the LAB *L. kimchii* isolated during traditional *pulque* fermentation. Screening of LAB was based in their ability to produce EPS from sucrose; this method resulted in the identification of two unique EPS-producing colony types. Structural characterization of these polymers including SEM during EPS production conditions, enzymatic and acid hydrolysis, as 1D- and 2D- ^1^H- and ^13^C-NMR allowed the detection of dextran and levan polymers produced by this LAB*.*

## Results and discussion

### Isolation and identification of EPS-producing LAB

**Figure 1:**
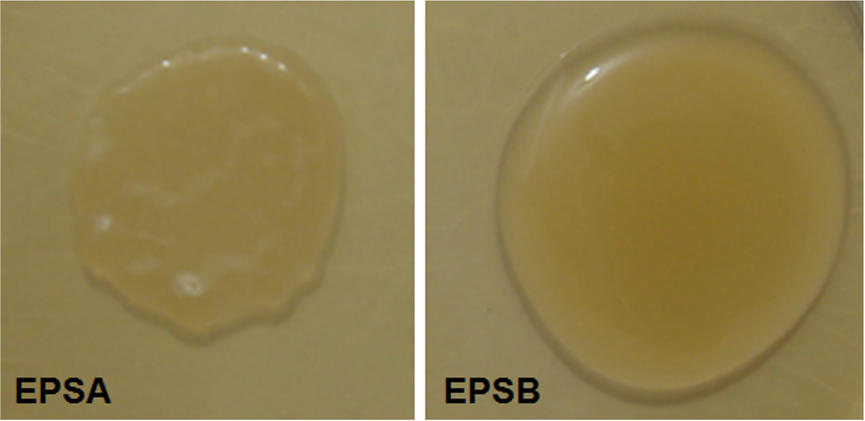
**Phenotypic traits of EPS produced by** ***pulque*** **isolated** ***Leuconostoc kimchii*** **strains.** Compact left colony or EPSA and creamy right colony or EPSB grown on APT plates supplemented with 20% sucrose.

The total EPS-producing LAB CFU/mL detected by growth on APTS plates was 50% in previously fermented *pulque*, 47% in *aguamiel*, 50.8% at T0, 70.4% at T3, and 50% at the end of the fermentation (T6), compared to the total LAB CFU/mL grown on APT plates. These results indicated a great abundance of EPS-producing LAB in *pulque*, *aguamiel*, and during the fermentation process. The visual analysis of purified EPS-producing colonies for unique morphology grown on APTS, allowed the identification of only two EPS colony phenotypes, leading to the conclusion that despite the high number of polymer-producing LAB, phenotypic diversity is low. The fastest growing colony of each EPS type was selected and designated as EPSA (compact colony morphology) and EPSB (creamy colony morphology) (Figure 1).

**Figure 2:**
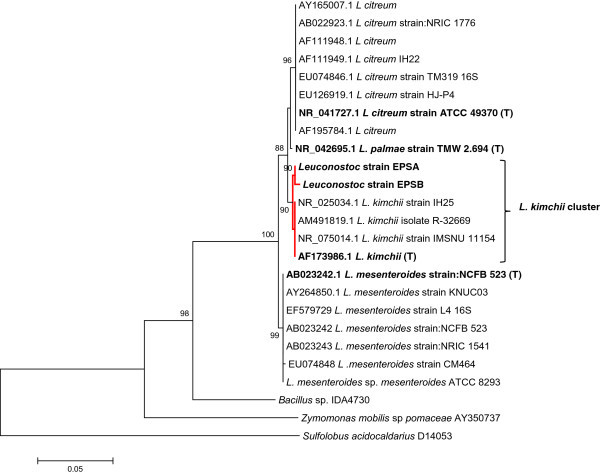
**Phylogenetic tree of 16S rDNA sequences of EPSA and EPSB isolates and 16S rDNA reference sequences.** Some 16S rDNA sequences of *L. citreum, L. palmae, L. kimchii*, and *L. mesenteroides* strains deposited in the GenBank database including type strains of each genus (bold) are included as references. Accession numbers of reference sequences are indicated. The 16S rDNA sequences of *Sulfolobus acidocaldarius* and *Zymomonas mobilis* are included as outgroups. The percentage of 1000 bootstrap samplings supporting each topological element in the neighbor-joining analysis is indicated. No values are given for groups with bootstrap values less than 80%. *L. kimchii* cluster including EPSA and EPSB sequences is highlighted.

Analysis of the 16S rDNA sequence of strains EPSA and EPSB in the non-redundant database of the NCBI archive database showed that the closest matching sequences found, corresponded to *L. kimchii* strain IMSNU 11154 (an isolated LAB from Korean *kimchi*)*, L. palmae* strain TMW 2.694, and diverse *L. citreum* 16S rDNA sequences in both cases. To precise identity of EPS isolates, a phylogenetic analysis was performed using several *Leuconostoc* 16S rDNA reference sequences retrieved from the GenBank database, such as *L. citreum, L. kimchii, L. mesenteroides,* and *L. palmae* including type strains*.* The resulting neighbor-joining tree clearly demonstrated that the 16S rDNA sequences of the EPSA and EPSB isolates were placed in a cluster in which only *L. kimchii* sequences were included. However, the 16S rDNA sequence from *L. palmae* strain: TMW 2.694, isolated from palm wine (Ehrmann et al. 2009), was placed in the closest single terminal node separated from the well-defined clusters of the 16S rDNA sequences from *L. kimchii*, *L. citreum*, and *L. mesenteroides* (Figure 2). The 16S rDNA sequences corresponding to the EPSA and EPSB *L. kimchii* strains isolated from *pulque* were deposited into the GenBank database and accession numbers KC424437 and KC424438 were assigned to strains EPSA and EPSB, respectively. *L. kimchii* strain IMSNU 11154 was originally isolated from traditional Korean *kimchi,* a traditional fermented vegetable food (Kim et al. 2000). Complete genome sequencing of the EPS-producing *L. kimchii* strain IMSNU 11154 was reported and demonstrated the presence of genes corresponding to GTFs capable to produce dextran from sucrose (Oh et al. 2010).

### Scanning electronic microscopy of EPS-producing LAB

**Figure 3:**
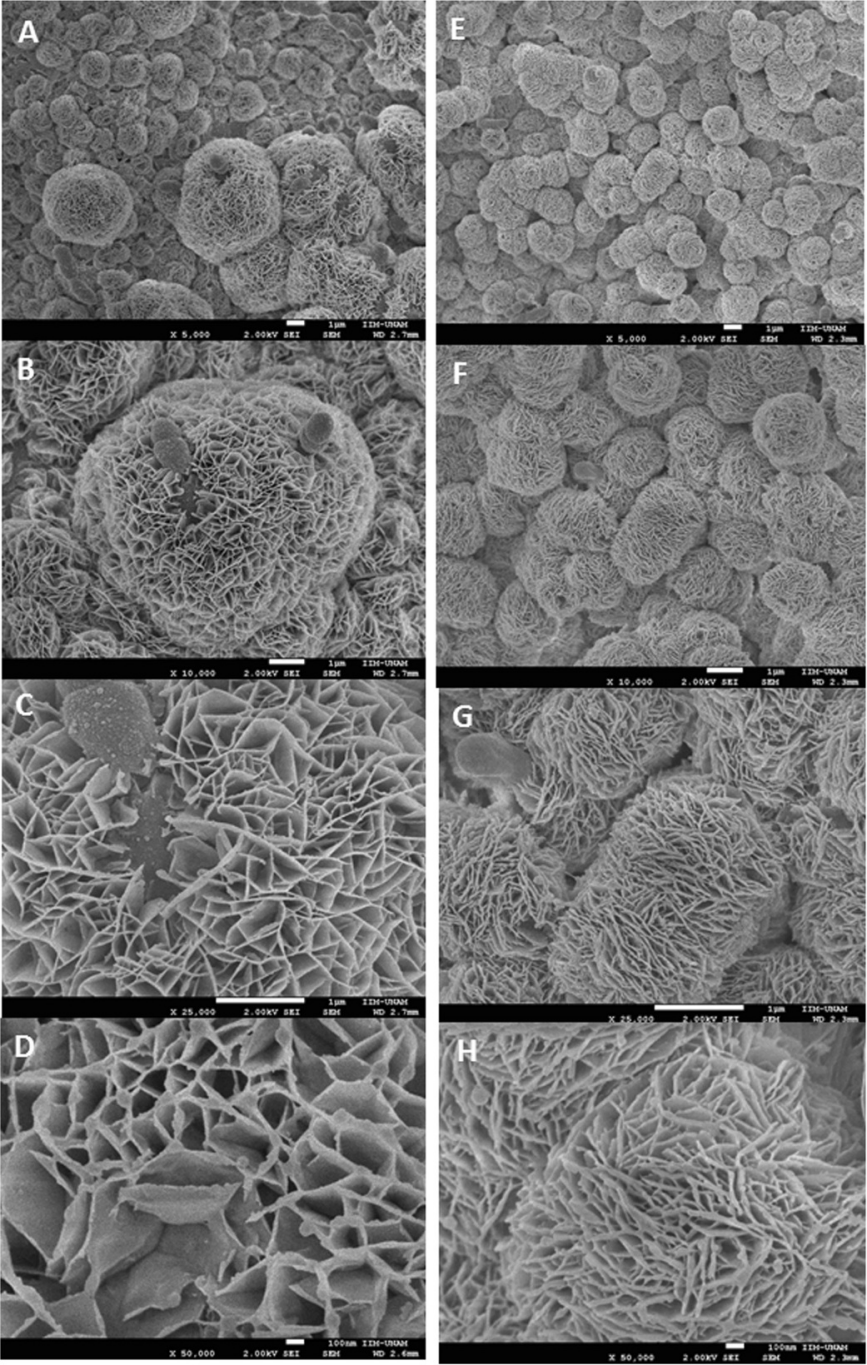
**Scanning electron microscopy analysis of EPS-producing** ***Leuconostoc kimchii.*** Images **A-D**, correspond to different fields of EPSA producing strain at 5 000X, 10 000X, 25 000X, and 50 000X, respectively. Images **E-H**, correspond to different fields of EPSB producing strain at 5 000X, 10 000X, 25 000X, and 50 000X, respectively.

The SEM observation of EPS-producing colonies of EPSA and EPSB growing on APTS plates showed the presence of porous -“cocoon-like” structures associated with the cells (cocci) in both strains. These structures surround the bacterial cells and have different sizes depending on the colony, with larger structures observed for EPSA (Figure 3). Although for both strains a surrounding hollow porous structure is observed, the polymer matrix produced by the EPSB strain appears more compact when compared to the EPSA polymer matrix. No other dextran produced by LAB has been previously analyzed by SEM, although a rather similar porous structure was previously reported for the dried cell-free insoluble dextran produced by *L. mesenteroides* NRRL B-1149 (Shukla et al. 2011).

### Evidence of glycosyltransferase activity

Total GTF activity was determined either in the cell culture supernatant or associated to the cell fraction of cultures for *L. kimchii* EPSA and EPSB isolates on APTS broth. For the EPSA isolate, a maximum of 3.2 U/L was observed in the supernatant after 6 h of fermentation, when the pH decreased to 4.4. GTF activity was also found to be associated with the cultures´s cell fraction showing a maximum activity of 1.8 U/L after 4 h. When the culture supernatant reached pH 5.5; the insoluble activity was approximately 36% of the total GTF activity produced by the cells. The total GTF activity was found higher in a similar analysis performed with the EPSB strain. In this case the soluble GTF activity reached 11.21 U/L after 6 h of fermentation when the culture supernatant pH was 4.6, while the cell-associated GTF activity reached a maximum of 5.57 U/L after 6 h, representing 33% of the total GTF activity. These culture conditions were selected to produce soluble and cell-associated polymers for structural characterization. It was also found that the isolated strains EPSA and EPSB showed higher residual total GTF activity in the temperature range of 30°C - 40°C, both in soluble and cell-associated fractions. Regarding the pH, both EPSA and EPSB-soluble fractions showed higher residual total GTF activity at pH 5.4, while both EPSA and EPSB cell-associated fractions showed higher residual total GTF activity at pH 6.0.

The GTF activity level associated only to the soluble or cell-associated fractions, or in both fractions ranging 0.8 to 2 U/mL was previously detected in several *Leuconostoc* and *Weissella* isolated strains from fermented sources (Bounaix et al. 2009; Vasileva et al. 2012). Our results showed that the GTF activity detected in the soluble fractions of *L. kimchii* EPSA and EPSB strains, was higher than the activity found in the cell-associated fractions was but low when compared with previous reports (Bounaix et al. 2010; Bounaix et al. 2009; Vasileva et al. 2012). EPS production by LAB from sucrose was reported that depends on diverse factors such as the cultivation conditions (aerobic, anaerobic and temperature) and media composition (liquid or solid media, rich media such as MRS or APT or mineral media supplemented with phosphate sources, tryptone or yeast extract) (Maina et al. 2008; Minervini et al. 2010). While further investigation is required to determine the fine enzymatic properties of GTF responsibles for soluble and cell-associated EPS production in both EPSA and EPSB *L. kimchii* isolates, to our knowledge, these results provide evidence that GTF is involved in EPS production by this LAB isolated from a traditional alcoholic fermented beverage for the first time. Previous studies involving both glucosyltransferase and fructosyltransferase characterization have been performed particularly in diverse EPS-producing *Leuconostoc, Weissella,* and *Lactobacillus* species isolated from fermented beverages, cereal doughs, and vegetables (Table 1).

**Table 1:** **EPS produced from sucrose by LAB isolated from traditional fermented products**

EPS type	EPS structure (producing LAB)	Source	Reference
Dextran	Linear backbone linked mainly in α-(1→6) D-Glc*p* with branching in α-(1→3) D-Glc*p* produced by a cell-associated GTF (*L. mesenteroides* IBT-PQ strain)	*Pulque* (fermented alcoholic beverage)	(Chellapandian et al. 1998)
Dextran^a^	Linear backbone linked mainly in α-(1→6) D-Glc*p* with branching in α-(1→2) (*L. mesenteroides*).	Palm wine	(Uzochukwu et al. 2001)
	Linear backbone linked mainly in α-(1→6) D-Glc*p* with branching in α-(1→3) with minor α-(1→4) linked branches (*L. dextranicum*).		
	Linear backbone linked mainly in α-(1→6) D-Glc*p* with branching in α-(1→3) (*Lactobacillus* spp. AW strain)		
Fructan	Inuline-like structure with β-(2→1) glycosidic linkages produced by a cell-associated fructosyltransferase (*L. citreum*)	*Pozol* (maize fermented dough)	(Olivares-Illana et al. 2002)
Dextran^a^	Dextran type I containing α-(1→2) D-Glc*p* and α-(1→3) linked branches (*L. citreum* VTT E-93497 strain).	Malting process	(Maina et al. 2008)
	Dextran type I containing few α-(1→3) linked branches (*W. confusa* VTT E-90392 strain)	Soured carrot mash	
Dextran	Dextran type I linked mainly in α-(1→6) D-Glc*p* with few α-(1→3) linked branches produced by soluble GTF (Several *L. mesenteroides* and *Weissella* spp isolates)	Traditional French wheat sourdough	(Bounaix et al. 2009)
	Dextran type I linked mainly in α-(1→6) D-Glc*p* with α-(1→2) linked branches produced by soluble GTF (several *L. mesenteroides* and *L. citreum* isolates)		
	Dextran type I linked mainly in α-(1→6) D-Glc*p* with high α-(1→3) linked branches produced by soluble GTF (Several *L. citreum* isolates)		
Dextran/levan mixture	Dextran type I linked mainly in α-(1→6) D-Glc*p* with high α-(1→3) linked branches produced by soluble and cell-associated GTF mixed with a levan (several *L. mesenteroides* isolates)	Traditional French wheat sourdough	(Bounaix et al. 2009)
Dextran	Dextran type I linked mainly in α-(1→6) D-Glc*p* with few α-(1→2) linked branches (*W. confusa* LBAE C39-2)	Traditional French wheat sourdough	(Amari et al. 2012)
Dextran^a^	Linear backbone linked mainly in α-(1→6) D-Glc*p* (*Lactobacillus curvatus* 69B2 strain and *Leuconostoc lactis* 95A strain)	Wheat sourdough	(Palomba et al. 2012)
Dextran	Linear backbone linked mainly in α-(1→6) D-Glc*p* with α-(1→3) linked branches produced by soluble and cell-associated GTF (*L. mesenteroides*)	Bulgarian fermented vegetables	(Vasileva et al. 2012)
Dextran/levan mixture	Dextran linked mainly in α-(1→6) D-Glc*p* (56%) and a levan (44%) (*L. mesenteroides* URE and Lm 17 strains)	Bulgarian fermented vegetables	(Vasileva et al. 2012)
Dextran^a^	Linear backbone linked mainly in α-(1→6) D-Glc*p* (*L. mesenteroides* and *W. confusa*)	*Kimchi* (traditional fermented vegetable food)	(Park et al. 2013)
Dextran	Linear backbone linked mainly in α-(1→6) D-Glc*p* with α-(1→2) and α-(1→3) linked branches produced by a soluble and cell-associated GTF (*L. kimchii* EPSA strain)	*Pulque*	This work
	Dextran type I containing few α-(1→3) linked branches produced by a soluble GTF (*L. kimchii* EPSB strain)		
Levan/dextran mixture	Polymer mixture composed by linear chains of (2→6)-linked β-D-fructofuranosyl residues with connections β-(2→6) (79%), and a dextran Type I (21%) produced by the cell-associated GTF fraction (*L. kimchii* EPSB strain)	*Pulque*	This work

^a^No information about GTF producing enzymes is provided.

### EPSA and EPSB characterization

#### Hydrolysis of soluble and cell-associated EPS fractions

**Figure 4:**
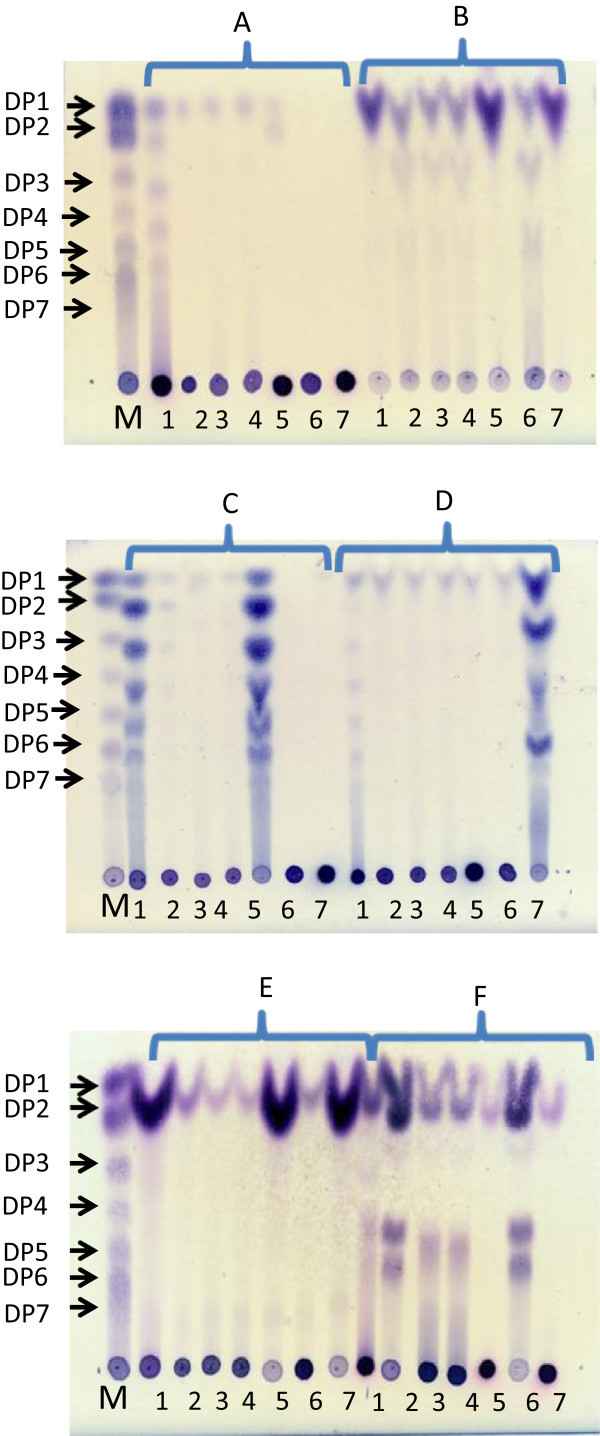
**Enzymatic and acid hydrolysis of EPSA and EPSB polymers.** 1. EPSB cell-associated fraction; 2. EPSB-soluble fraction; 3. EPSA cell-associated fraction; 4. EPSA-soluble fraction; 5. Levan from *B. subtilis*; 6. Dextran from *L. mesenteroides* B-512; 7. Inulin from *L. citreum.* M. Glucose + fructose + maltodextrins standards; **A**. Non-treated samples; **B**. Acid hydrolysis treatment; **C**. Endolevanase treatment; **D**. Endoinulinase treatment; **E**. Fructozyme^®^ treatment; **F**. Dextranase treatment. DP = Degree of polymerization.

Enzymatic hydrolysis assays of the cell-associated and soluble EPSA and EPSB polymer samples showed that polymers produced by the EPSA-soluble and cell-associated GTF were hydrolyzed only by dextranase but not by endolevanase, inulinase or Fructozyme^®^. These results indicated the presence of a dextran with α-(1→6) D-Glc*p* linkages (Figure 4), similar to dextrans that result from GTF synthesis. Similarly, the polymer produced by the EPSB-soluble fraction was also only hydrolyzed by dextranase, demonstrating the presence of α-(1→6) D-Glc*p* linkages as in dextran. Nevertheless, the polymer produced by the EPSB cell-associated fraction was degraded by dextranase, endolevanase and Fructozyme^®^ but not by inulinase, indicating the presence of a polymer mixture composed of a linear dextran and a levan. Furthermore, acid treatment of EPS with H_2_SO_4_ yielded hydrolysis giving the expected monomers.

#### ^***1***^***H- and***^***13***^***C-NMR analysis of soluble and cell-associated EPSA and EPSB fractions***

As enzymatic hydrolysis demonstrated that soluble and cell-associated fractions of EPSA are the same polymer, only the ^1^H- and ^13^C-NMR spectra of the EPSA-soluble fraction was determined, whereas for the soluble and cell-associated polymers of *L. kimchii* EPSB strain both spectra were obtained. Resultant NMR spectra were compared with those previously reported for diverse polymers produced by several *L. mesenteroides*, *L. citreum*, and *Weissella* sp. strains (Colson et al. 1974; Seymour et al. 1976; Seymour et al. 1979; Shimamura et al. 1987; Uzochukwu et al. 2001; Uzochukwu et al. 2002; Maina et al. 2008; Bounaix et al. 2009; Vasileva et al. 2012).

**Figure 5:**
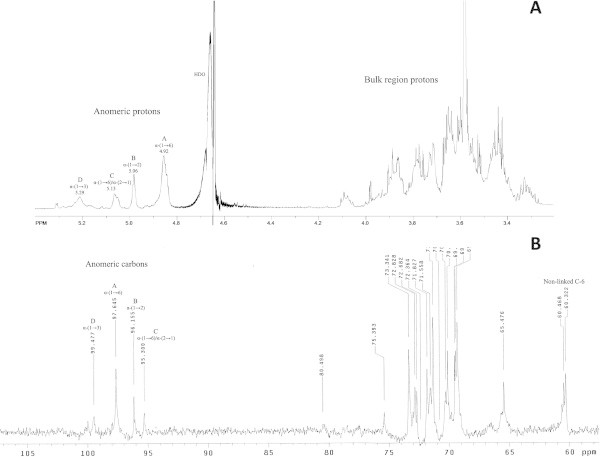
**NMR spectra of EPSA soluble fraction. (A)** ^1^H-NMR spectrum. **(B)** ^13^C-NMR spectrum. Anomeric protons are labeled A–D according to the increasing chemical shifts.

Comparison of the NMR results with previously reported spectra for dextrans produced during palm wine fermentation (Uzochukwu et al. 2002) revealed that EPSA is a dextran with a linear backbone of linked α-(1→6) D-glucopyranosyl units in the main chain confirming enzymatic hydrolysis assay results, but with α-(1→2) and α-(1→3) D-Glc*p* linked branches. The ^1^H-NMR spectra showed four anomeric proton signals at δ 4.92, 5.06, 5.13, and 5.29 labeled A-D, respectively (Figure 5A) corresponding to the α-(1→6) D-Glc*p*, the α-(1→2) branching D-Glc*p*, the 2,6 di-*O*-substituted α-D-Glc*p,* and the α-(1→3) D-Glc*p* units, respectively. The relative intensities of the A-D peaks were 15.30%, 12.41%, 16.86%, and 55.43%, respectively, resulting proportional to the degree of branching. HSQC of EPSA (Additional file 1) showed their attachment to the anomeric carbons at δ 97.64, 96.15, 95.30, and 99.47 corresponding respectively to ^1^H anomeric signals at δ 4.92, 5.06, 5.13, and 5.29 (Figure 5B). These signals characteristically served as the starting point for the analysis of the ^1^H–^1^H COSY and TOCSY experiments for connectivities within the spin system.

The C-H correlation spectrum (HSQC) indicated that ^13^C resonances at δ 65.48 split into two peaks (Additional file 1). The correlation peak showed the ^1^H resonance at δ 3.70 and δ 3.93. The other ^13^C peaks at δ 60.32, 60.47, and 60.65 showed their protons as a multiplet at δ 3.78 and δ 3.66 which corresponded to non-linked C-6. The previous characterization of dextrans from fermented palm wine showed that its backbone contains a bound C-6 with a chemical shift at δ 66.6 (Uzochukwu et al. 2002), whereas for EPSA polymer it was found at δ 65.48, resulting farthest downfield than a free C-6 at δ 61.4 (δ 60.32, 60.47, and 60.65 for EPSA polymer). Finally, the long-range correlation between H-3 (δ 3.81) and the anomeric carbon at δ 99.47 of EPSA polymer in the HMBC spectrum allowed to confirm the presence of α-(1→3)-linked branches (Additional file 1).

Furthermore, in the δ 68–74 region of the ^13^C-NMR spectrum of EPSA, were found characteristic branching signals for linked C-2 and C-3 at branching points, which split in three peaks found in δ 71.83, 71.56, and 71.31; and in the region at δ 69.53, 69.46, and 69.31, respectively (Figure 5b). These data were consistent with previous results of spectra for the series of dextrans B1254, B1355S, and B1099L (Seymour et al. 1976). The split in the free C-6 around δ 60-61 represents C-6 in different chemical environments with two possible sources of branch-terminating residues: α-(1→2) and α-(1→3) branching. Free C-6 signals in EPSA polymer were detected at δ 60.32, 60.47, and 60.65, respectively and were associated with branching at C-2 and C-3, respectively, indicating that in EPSA polymer the linkage is present both as branch point and intra chain linkages as reported previously for palm wine dextran (Uzochukwu et al. 2002).

**Figure 6:**
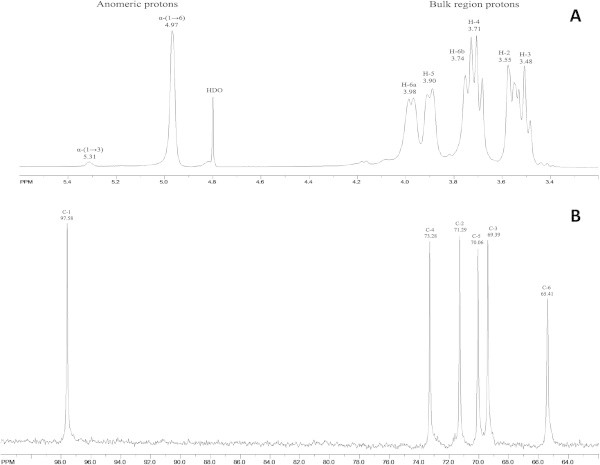
**RMN spectra of EPSB-soluble fraction. (A)** ^1^H-NMR spectrum. **(B)** ^13^C-NMR spectrum.

NMR spectra results for EPSB polymers corroborated that its soluble fraction is a dextran with a linear backbone containing consecutive α-(1→6)-linked D-glucopyranosyl units with few α-(1→3)-linked branches, structure characteristic of class 1 dextrans (Maina et al. 2008; Amari et al. 2012). The ^1^H-NMR spectrum for EPSB-soluble fraction (Figure 6A), revealed anomeric signals characteristic of glucosyl residues linked through α-(1→6) (98.5%) and α-(1→3) (1.5%) linkages appeared at δ 4.97 and δ 5.3, respectively. The other protons appeared from δ 3.4 to 4.1: δ = 3.55 (dd, 1H, *J* = 3.2 Hz, *J* = 9.8 Hz, H-2), 3.48 (t, 1H, *J* = 9.2 Hz, *J* = 9.6 Hz, H-3), 3.71 (t, 1H, *J* = 9.2 Hz, *J* = 9.6 Hz, H-4), 3.90 (d, *J* = 8.4, 1H, H-5), 3.74 (d, *J* = 7.48, H-6a), 3.98 (d, *J* = 9.76, 1H, H-6b). The ^13^C-NMR spectrum (Figure 6B) shows six signals, among them, those appearing at δ 97.56 and δ 65.41, corresponding to C-1 and C-6, which are involved in α-(1 → 6) linkages. The linkage carbon resonance at δ 65.41 showed two correlation peaks (δ 3.74 and δ 3.98) in the HSQC spectrum (Additional file 1). The four remaining signals observed at δ 73.28, 70.06, 69.39, and 71.29 corresponded to C-4, C-5, C-3, and C-2, respectively. Full assignments of the proton and carbon resonances were secured from the TOCSY, COSY, NOESY, and HMBC data.

These results are consistent with previous reported ^1^H- and ^13^C-NMR data for the dextran produced by *W. confusa* C39-2 strain with 97.6% α-(1 → 6) and 2.4% α-(1 → 3) linkages, respectively (Bounaix et al. 2009). The structure of the soluble EPSB dextran also resembled that of dextrans produced by *W. confusa* VTT E-90392 (0DSM 20194) (Maina et al. 2008) and *W. cibaria* CMGDEX3 (Ahmed et al. 2012).

In other hand, the ^1^H- and ^13^C-NMR chemical shifts observed suggest that the polymer from the cell-associated fraction of isolated strain EPSB is a mixture that consist of a levan (79%) composed of linear chains of (2→6)-linked β-D-fructofuranosyl residues with β-(2→6) linkages, and a polymer of class 1 dextran (21%). The assignments of methylene and methine carbons were determined by a DEPT analysis (Additional file 1). The ^13^C-NMR spectrum gave three upfield resonances which were methylene (δ_C_ 59.83, 63.32, 65.46) while the remaining resonances were methines and one quaternary carbon (δ_C_ 104.13).

**Figure 7:**
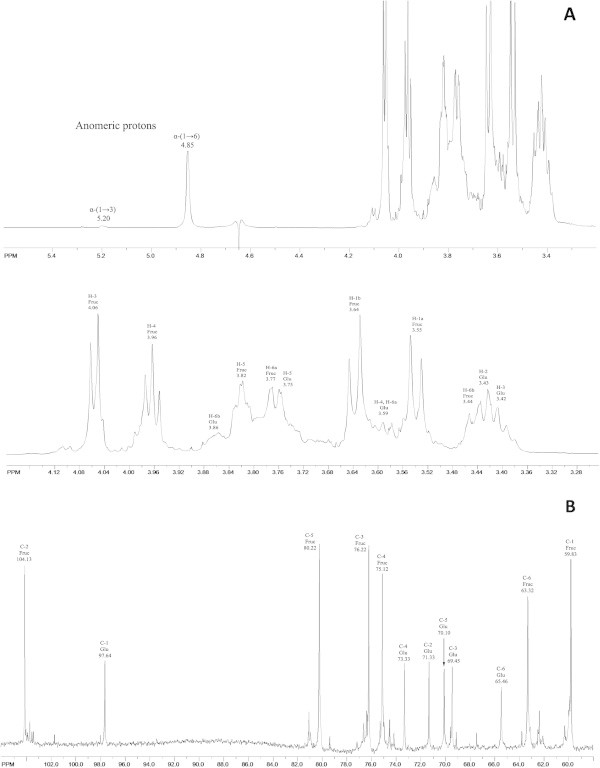
**RMN spectra of EPSB-soluble and cell-associated fractions. (A)** Full ^1^H NMR spectrum (upper graph) and region between 4.2 and 3.1 nm (bottom graph). **(B)** ^13^C-NMR spectrum.

The ^1^H-NMR spectrum showed intense signals associated to the fructose moieties in the levan fraction (Figure 7A), which were detected at δ 3.77 (d, *J* =12.6 Hz, H-6a), 3.44 (d, *J* =11.2 Hz, H-6b), δ 3.55 (d, *J* = 11.9 Hz, H-1a), δ 3.64 (d, *J* = 11.9 Hz, H-1b), δ 3.82 (ddd, *J* = 2.8 Hz, *J* = 7.7 Hz, *J* = 7.7 Hz, H-5), δ 3.96 (t, *J* = 7.7 Hz, *J* = 8.4 Hz, H-4), δ 4.06 (d, *J* = 8.4 Hz, H-3). The COSY spectrum was used to detect cross peaks between H-3/H-4, H-4/H-5, and H-5/H-6. The HMBC spectrum (Additional file 1) showed the presence of cross peaks between H3/C4, H4/C6, H4/C3, H1/C3, and H1/C2 as it was reported previously (Dahech et al. 2013). This result allowed to confirm the presence of β-(2→6) linkage between two fructofuranosyl moieties, but the β-(2→6) linkage was confirmed only by the presence of a downfield shifted signal at δ 63.32 (C-6) in the ^13^C-NMR spectrum as reported previously (Tomašić et al. 1978). It has been shown that normally glycosylation induces a downfield shift of δ 4-10 (Newbrun and Baker 1968). In the ^13^C-NMR spectrum (Figure 7B) of the EPSB cell-associated polymer were detected six main resonance shifts at δ 59.83 (C-1), δ 63.32 (C-6), δ 75.12 (C-4), δ 76.22 (C-3), δ 80.22 (C-5), and δ 104.13 (C-2) relating to a quaternary anomeric carbon, corresponding to levan. These results are also similar to those observed previously in levan produced by *L. mesenteroides* B-512 F (Morales-Arrieta et al. 2006) and *Streptococcus mutans* (Shimamura et al. 1987).

According to 1D- and 2D-NMR analysis, the second polymer in the mixture corresponded to dextran. The chemical shifts for the 6 carbons of the dextran were linked to those of the seven hydrogens as determining by using HSQC (Additional file 1). The characteristically downfield anomeric carbon (δ 97.64) and anomeric hydrogen (d, δ 4.85, *J* = 1.4 Hz) was considered as the starting point for the analysis of the ^1^H-^1^H COSY (Additional file 1) for connectivity within the spin system. The two diasterotopic H-6 hydrogens at δ 3.86 (m) and at δ 3.59 (m) in the ^1^H spectrum were also useful markers and attached to the carbon at δ 65.46 in the ^13^C spectrum. The other protons appeared in the spectrum at: δ = 3.43 (H-2), 3.42 (H-3), 3.59 (H-4 and H-6a), 3.75 (H-5), 3.86 (H-6b). In the ^13^C-NMR spectrum, the peaks corresponding to individual carbons were identified at δ C-4 (73.33), C-2 (71.33), C-5 (70.10), C-3 (69.45), and C-6 (65.46), respectively (Figure 7B).

Polymers synthesized by GTF from sucrose by diverse LAB isolated from different traditional fermented sources include linear and branched dextrans, dextran and levan mixtures, and also inulin like polymers, indicating a high natural diversity of these EPS, particularly interesting considering the wide geographical distribution of these fermented products: palm wine from Africa, cereal sourdoughs and fermented vegetables from Europe, traditional *kimchi* from Korea, and maize sourdough and a fermented *pulque* beverage from Mexico (Table 1). The production of a dextran and levan polymers mixture was previously detected in the cell-associated fraction of cultures on MRS-sucrose of *L. mesenteroides* G15 strain isolated from a French cereal sourdough as revealed by ^13^C-NMR spectroscopy (Bounaix et al. 2009). Interestingly, detection of genes coding for glucosyltransferase and enzymes demonstrated their respective presence in isolated *L. mesenteroides* strains from Bulgarian fermented vegetables, but activity of putative fructosyltransferase was detected only in the presence of raffinose instead sucrose (Vasileva et al. 2012). Additionally, the production of an inulin-like polymer with β-(2→1) glycosidic linkages, was demonstrated by ^13^C-NMR in a cell-associated fructosyltrasferase in cultures containing 20 g/L sucrose of *L. citreum* strain CW isolated from *pozol*, a Mexican fermented maize sourdough (Olivares-Illana et al. 2002) (Table 1).

Several polymers described in Table 1 have relevant potential applications, as it has been proposed that branched dextrans possess prebiotic properties, demonstrating that these polymers and their oligodextrans may be substrates for butyric acid production by intestinal microbiota. Additionally, highly linear dextrans are considered highly soluble and were proposed to confer viscosity properties associated to their molecular weight (Maina et al. 2008). Furthermore, levans were shown to exhibit prebiotic effects, attracting attention for its antitumor properties, cholesterol-lowering properties and application such eco-friendly adhesive and as a promising bio-thickener in food industry (Patel et al. 2011). *Pulque* beverage is consumed directly from the fermentation vessel without further antimicrobial treatment such as pasteurization or filtration, as a consequence, living microorganisms are consumed. Additionally, *pulque* has been traditionally considered has a healthy beverage due to its nutrient content and used as a natural medicine to control several diseases (Escalante et al. 2012). Even though proposed healthy effects have been limited to traditional pharmacopoeia, further evidence on the possible probiotic effect of LAB involved in the fermentation process as the possible prebiotic activity of dextran or dextran and levan mixture produced by LAB such *L. kimchii* EPSA and EPSB strains requires further investigation.

## Conclusions

EPSA and EPSB producing strains of *L. kimchii* isolated from traditional Mexican *pulque* and their polymers described in this contribution are to our knowledge the first study reporting the production and characterization of EPS produced by this LAB isolated from traditional fermented sources. *L. kimchii* was previously identified as the main LAB present in agave sap *aguamiel* and during the first hours of *pulque* fermentation. Although further studies are required to provide additional information concerning the enzymatic properties of the GTF responsibles for the synthesis of dextrans and levan by *L. kimchii* EPSA and EPSB strains, results presented in this contribution are highly relevant for *pulque* microbiology. In effect, while *L. mesenteroides* has traditionally been considered the main bacteria responsible for polymer production during *pulque* fermentation, this study provides new evidence regarding the diversity of EPS produced by LAB involved in the production of this traditional Mexican beverage.

## Methods

### *Aguamiel*and *pulque*sampling

Fresh *aguamiel* and fermented *pulque* were collected from the town of Hiutzilac, State of Morelos, Mexico (Central Mexico Plateau) and transported to the laboratory, where controlled fermentations were performed by the addition of fresh sap to previously fermented *pulque* as described before (Escalante et al. 2008). Samples of *aguamiel*, after inoculation (mixing of sap with previously fermented *pulque*) (sample T0) and after 3 and 6 h of fermentation, were serially diluted in 0.1% tryptone water (DIFCO), and aliquots of 0.1 mL were plated on APT-agar (DIFCO) and APT-agar supplemented with 20% (w/v) sucrose (APTS). Plates were then incubated at 30°C for 24 h to determine the total count of LAB and EPS-producing LAB, respectively.

### EPS-producing LAB isolation and identification

An average of 100 colonies of EPS-producing LAB from representative plates of *aguamiel* and each *pulque* sample were isolated, Gram stained, observed with a light microscope to verify colony purity, and tested for catalase activity. The purified isolated colonies were grown on APTS plates (4 colonies per plate), where the colony morphology and size were visually screened. Fast growing colonies were selected and visually screened for unique EPS-producing phenotypes resulting in the identification of two distinct EPS-producing colonies designated as EPSA and EPSB. Finally, selected isolates were conserved in 50% glycerol at -70°C and identified by 16S rDNA sequencing as described previously (Escalante et al. 2004; Escalante et al. 2008) by using fd1 + rd1 primer set resulting in the amplification of the entire 16S rRNA gene (Weisburg et al. 1991). Comparison was performed against the non-redundant nucleotide database using the online nucleotide BLAST application in the NCBI homepage. To corroborate their molecular identity, a phylogenetic tree was constructed using MEGA 6 software (Tamura et al. 2013) including reference and type 16S rDNA sequences retrieved from the NCBI database.

### Scanning electron microscopy analysis of EPS-producing LAB

EPS-producing LAB were grown on APTS plates until a viscous morphology was observed. Collodion-coated electron microscope grids were placed on growing colonies, and maintained for four additional days at room temperature to promote LAB adhesion to the grid. The grids were then lifted from the APTS plates and placed on a porcelain spot dish for further processing. Fixation was performed by 1 h of exposure to 1% paraformaldehyde and 2.5% glutaraldehyde in pH 7.4, 0.1 M phosphate buffer, followed by washing with the same buffer, post-fixing with 1% osmium tetroxide in buffer for 30 min and dehydration with graded alcohols. Throughout the procedure, special care was taken when changing solutions to avoid EPS dispersion and damage to the grid surface. The samples were then dried under critical CO_2_ (Sandri-780; Tousimis), gold coated (JFC-110 ion sputter; JEOL), and observed with a JEOL JSM-7600 F Field Emission SEM.

### Growth kinetics of EPS-producing strains

Flask cultures were performed in 50 mL of APT broth at 30°C and 150 rpm to define optimum growth conditions for selected LAB isolates and to achieve adequate enzyme activities and EPS production. The OD_600nm_ was determined and adjusted to 0.2 before inoculation in a 2.8 L flask with 560 mL of APT broth and incubation for 6 – 8 h at 30°C and 150 rpm. Microbial growth was followed by the OD_600nm_ measurements and the pH was monitored each hour.

### GTF activity assay

GTF activity was determined in cell-free supernatants and in harvested cell fractions by measuring the release of reducing sugars using the DNS technique (Sumner and Howell 1935) in the presence of 10% (w/v) sucrose in 100 mM, pH 5.4 acetate buffer. Incubation was performed in an Eppendorf Thermomixer Comfort device (Eppendorf, Hamburg) at 30°C. One activity unit (U) of total GTF activity is defined as the amount of enzyme producing 1 μmol of glucose per minute from GTF activity under the assay reaction conditions (Morales-Arrieta et al. 2006). Protein concentration was determined by the Bradford method (Bradford 1976), using the Bio-Rad reagent and BSA (BioRad) as standards.

### *In vitro*EPS production

EPS were produced from the supernatant and cell-associated enzyme fractions. For this purpose, the soluble GTF fraction was precipitated from the supernatant cultures by the addition of one volume of 50% (w/v) polyethylene glycol 5000 and centrifugation (7,000 × *g*, 5 min at 4°C). The pellet of cells containing the cell-associated GTF fraction, was washed three times and resuspended in a minimal volume of 0.1 mM, pH 5.4 acetate buffer (Quirasco et al. 1999). Polymer was produced in both fractions in the same buffer conditions containing 10% sucrose in final volume of 600 μL and incubated at 30°C and 100 rpm for 14 h (New Brunswick Scientific G24 shaker). The polymers produced from the cell-associated fraction were centrifuged as above to separate them from the cell whereas polymer produced from the supernatant fraction was precipitated by the addition of one volume of absolute ethanol and harvested by centrifugation (2,367 × *g*, 5 min at 4°C). Both polymers were resuspended in a minimum volume of distilled water and dialyzed against distilled water, eluted in a cellulose membrane (Mw cutoff of 12,400 Da SIGMA-ALDRICH), and dried in a LABCONO FreeZone 5.4 freeze-dryer.

### Hydrolysis of EPS

Soluble and cell-associated ESPA and EPSB polymer fractions were subjected to enzyme hydrolysis with Fructozyme L^®^ (kindly provided by Novozymes), a commercial enzymatic preparation obtained from *Aspergillus niger* combining endo- and exoinulinase activities toward β-(2→1) and endo-β-(2→6) D-Fru*p* linkages, in pH 5, 0.05 M, acetate buffer, at 60°C; Dextranase (Enzimas y Productos Químicos S.A. de C.V., Mexico City), which hydrolyses α-(1→6) D-Glc*p* linkages, in 0.05 M acetate buffer, pH 5, at 50°C; recombinant *E. coli* (*levB*) endolevanase from *Bacillus licheniformis*, which degrades endo-β-(2→6) D-Fru*p* linkages (kindly provided by Dr. A. López-Munguía) in pH 6, 50 mM potassium phosphate buffer at 37°C; and endoinulinase Novozym^®^ 960 (Batch KNN00120 from Novozymes), which degrades endo-β-(2→6) D-Fru*p* linkages, in pH 6, 50 mM potassium phosphate buffer at 60°C. Acid hydrolysis was performed with H_2_SO_4_ 5% (v/v) at 95°C, 1 hr.

Enzymatic and acid hydrolysis assays were performed in a volume of 0.5 mL containing 2% w/v of EPS and were incubated for 12 h. Inulin from *L. citreum*, levan of *B. subtilis* (Ortiz-Soto et al. 2004) and dextran of *L. mesenteroides* B-512 PM 69,000 Da (Sigma) were used as controls. Hydrolyzed samples were analyzed by TLC using pre-coated TLC-sheets of Alugram^®^ Xtra Sil G/UV254 with a mobile phase of 11:3:11:1 acetic acid:chloroform:ethanol:water mixture. The spots of products were detected by spraying with an alcoholic solution of α-naphthol and sulfuric acid, followed by heating at 120°C for 3 min.

### ^1^H- and ^13^C-NMR analysis of EPS

The freeze-dried samples of each polymer were dissolved in 1.0 mL of D_2_O (Cambridge Isotope Laboratories, Inc.). NMR spectrum of soluble EPSA polymer was recorded on an Advance 700 MHz spectrometer (Varian) operating at 700 MHz for ^1^H-NMR and 175 MHz for ^13^C-NMR. NMR spectra of both EPSB fractions were recorded on an Advance 400 MHz spectrometer (Varian) operating at 400 MHz for ^1^H-NMR and 100 MHz for ^13^C-NMR. All measurements were performed at room temperature and were obtained using a 1.0 s relaxation delay. The data were acquired and processed using VNMRJ 2.0 software. Chemical shifts are listed in parts per million (ppm) and were made on the basis of ^1^H-^1^H COSY, ^1^H-^1^H TOCSY, NOESY, DEPT, HSQC, and HMBC spectral analysis (Additional file 1).

## Electronic supplementary material

Additional file 1: **2-D NMR analysis of EPSA and EPSB polymers.** ^1^H-^1^H COSY, ^1^H-1H TOCSY, NOESY, HSQC, and HMBC spectral analysis of EPSA and EPSB-soluble fractions and DEPT, ^1^ H- ^1^H COSY, ^1^H-^1^H TOCSY, NOESY, HSQC, and HMBC spectral analysis of EPSB-soluble and cell-associated fractions. (PDF 1 MB)

## Abbreviations

EPS: Extracellular polysaccharides
LAB: Lactic acid bacteria
NMR: Nuclear magnetic resonance
SEM: Scanning electron microscopy
GTF: Glycosyltransferase(s).
