# Supplementary material for: Screening and characterization of extracellular polysaccharides produced by Leuconostoc kimchii isolated from traditional fermented pulque beverage
Source: Springerplus. 2014 Oct 7;3:583. doi: 10.1186/2193-1801-3-583 (PMC4194309; doi:10.1186/2193-1801-3-583)
Supplement: Supplementary file 1 — Additional file 1: 2-D NMR analysis of EPSA and EPSB polymers. 1H-1H COSY, 1H-1H TOCSY, NOESY, HSQC, and HMBC spectral analysis of EPSA and EPSB-soluble fractions and DEPT, 1 H- 1H COSY, 1H-1H TOCSY, NOESY, HSQC, and HMBC spectral analysis of EPSB-soluble and cell-associated fractions. (PDF 1 MB) [file 40064_2014_1285_MOESM1_ESM.pdf]

^1H-^1H COSY, ^1H-^1H TOCSY, NOESY , HSQC and HMBC spectral analysis of EPSA-soluble fraction

COSY spectrum of EPSA-soluble fraction

NOESY spectrum of EPSA-soluble fraction

TOCSY spectrum of EPSA-soluble fraction

HSQC spectrum of EPSA-soluble fraction

HMBC spectrum of EPSA-soluble fraction

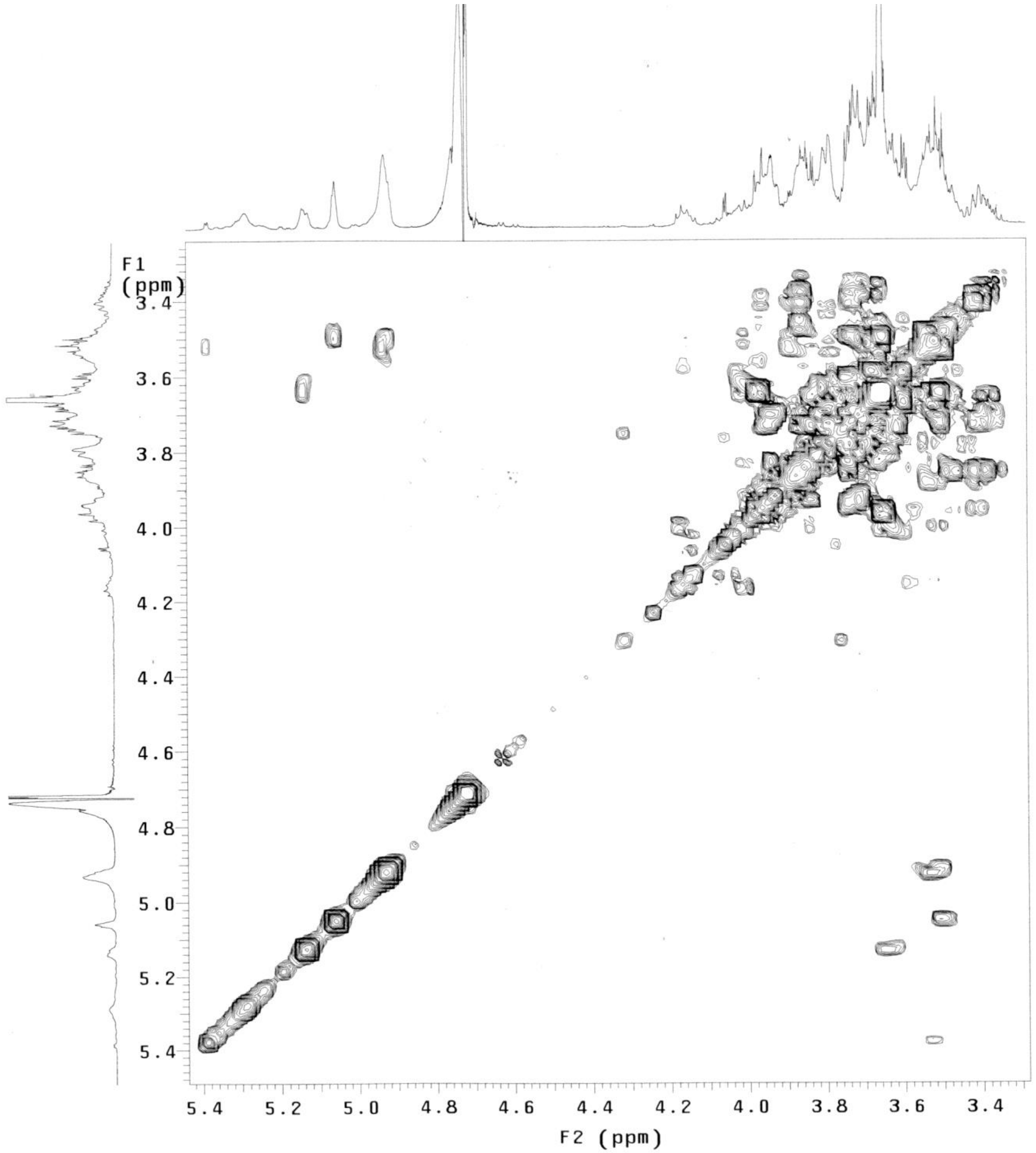

COSY spectrum of EPSA-soluble fraction

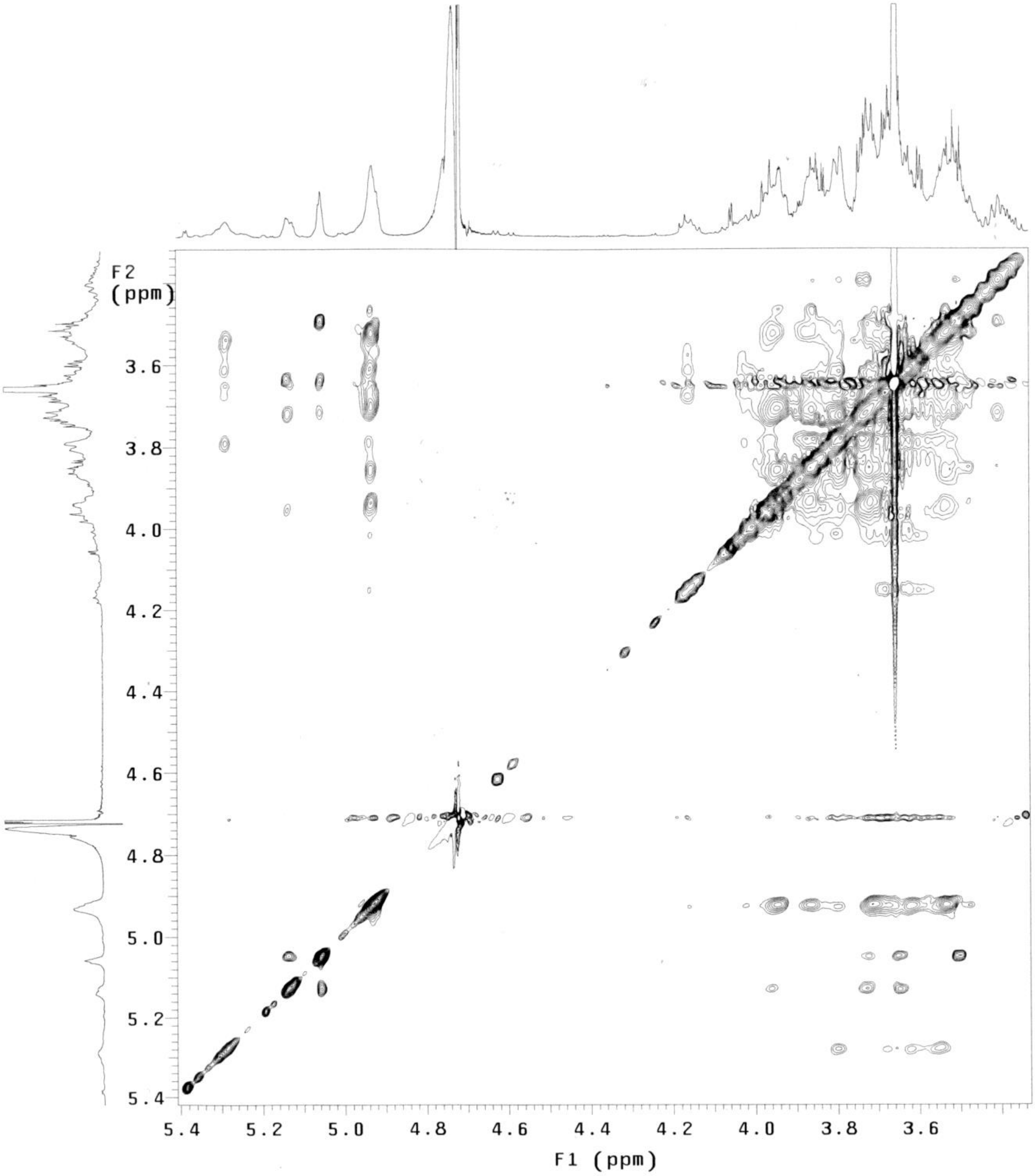

NOESY spectrum of EPSA-soluble fraction

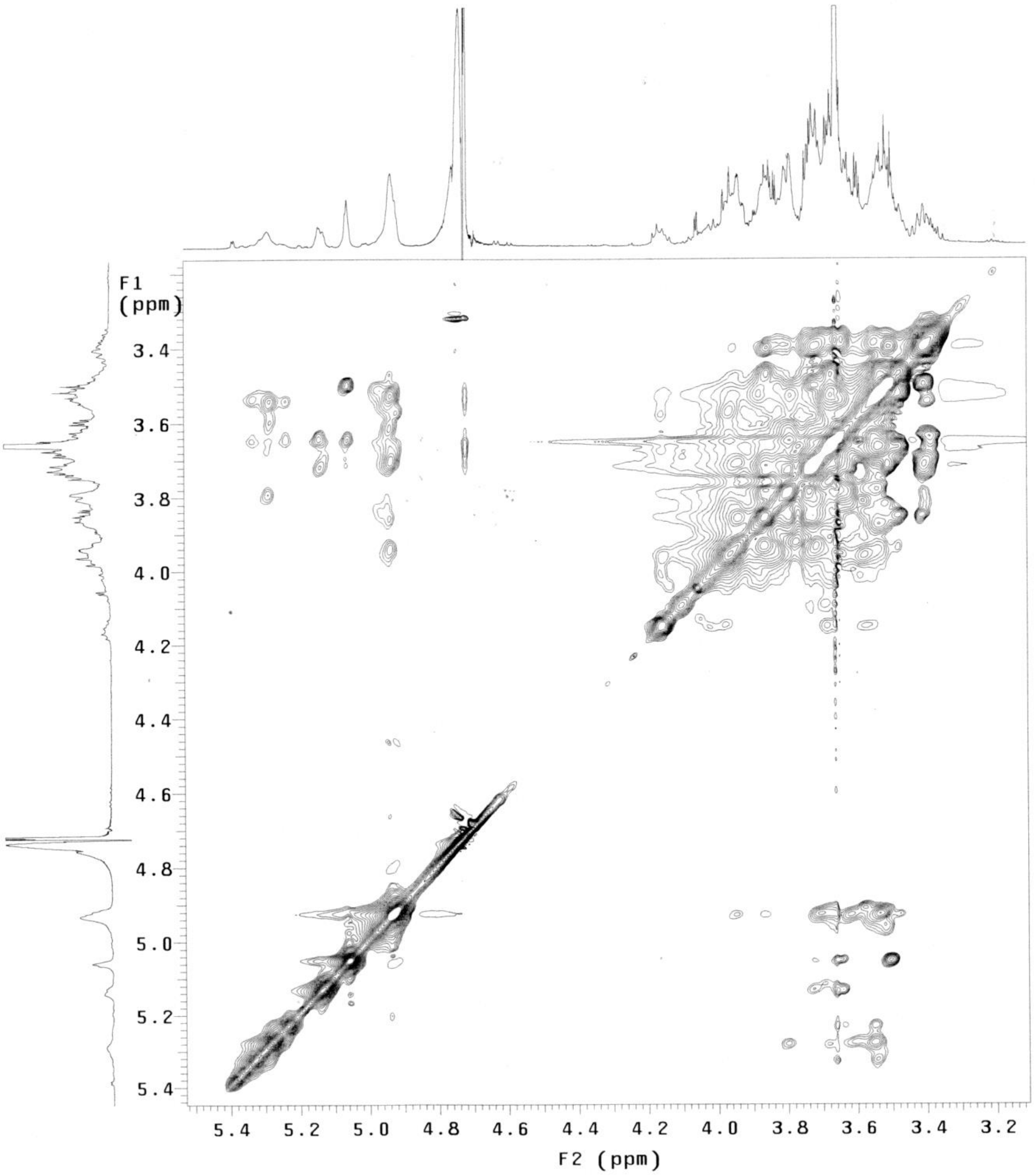

TOCSY spectrum of EPSA-soluble fraction

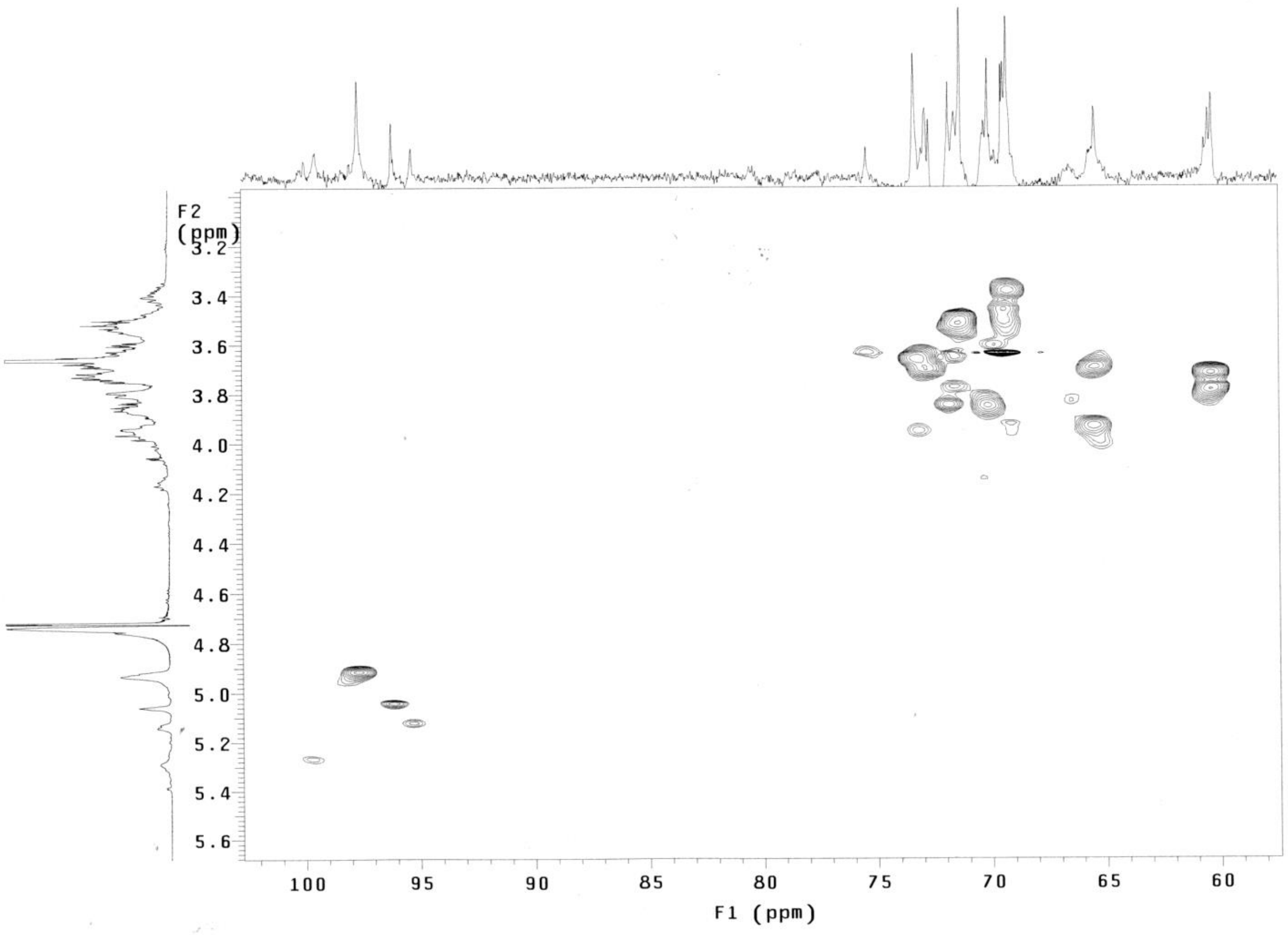

HSQC spectrum of EPSA-soluble fraction

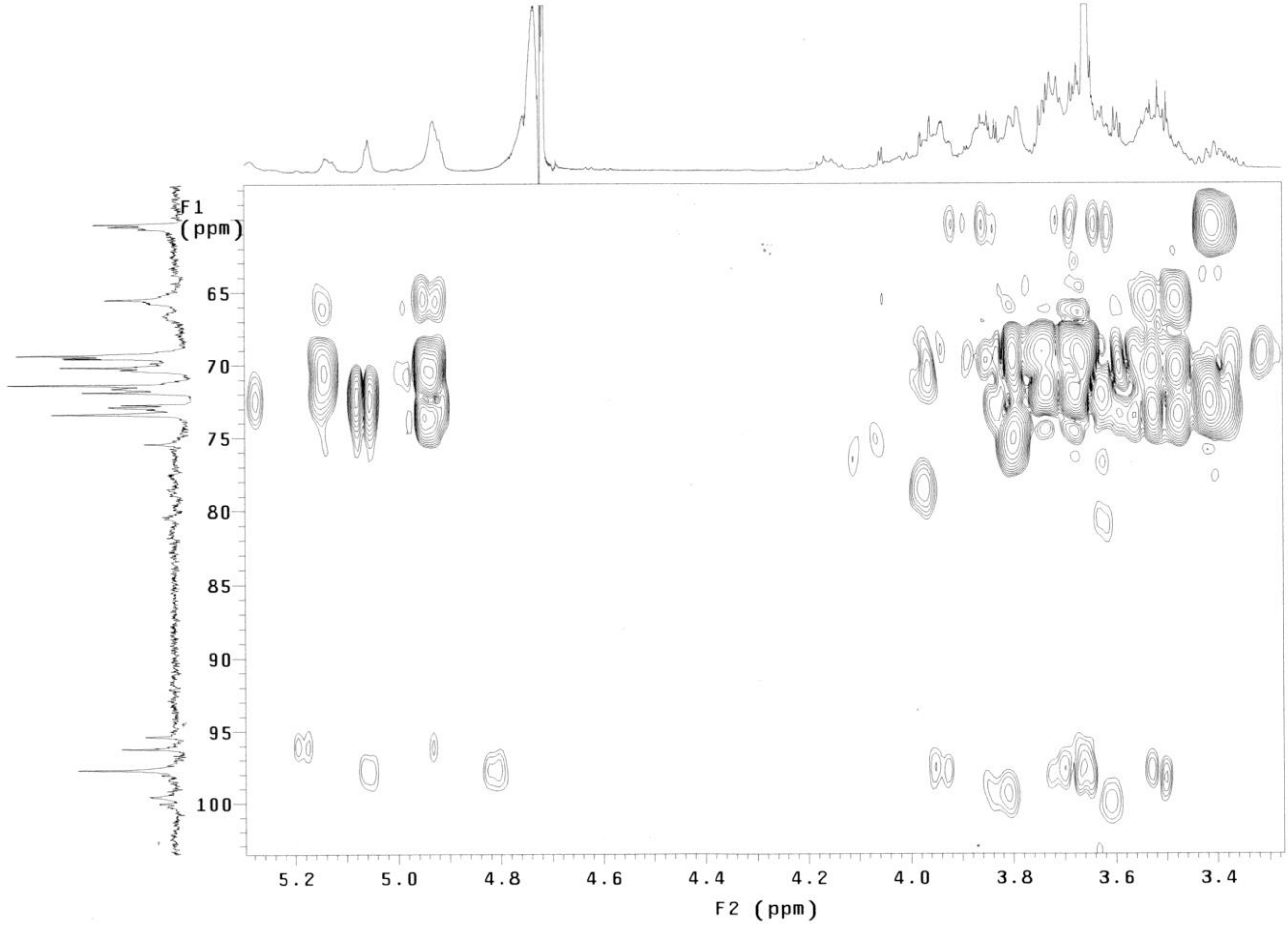

HMBC spectrum of EPSA-soluble fraction

^{1}H-^{1}H COSY, ^{1}H-^{1}H TOCSY, NOESY , HSQC and HMBC spectral analysis of EPSB-soluble fraction

COSY spectrum of EPSB-soluble fraction

NOESY spectrum of EPSB-soluble fraction

TOCSY spectrum of EPSB-soluble fraction

HSQC spectrum of EPSB-soluble fraction

HMBC spectrum of EPSB-soluble fraction

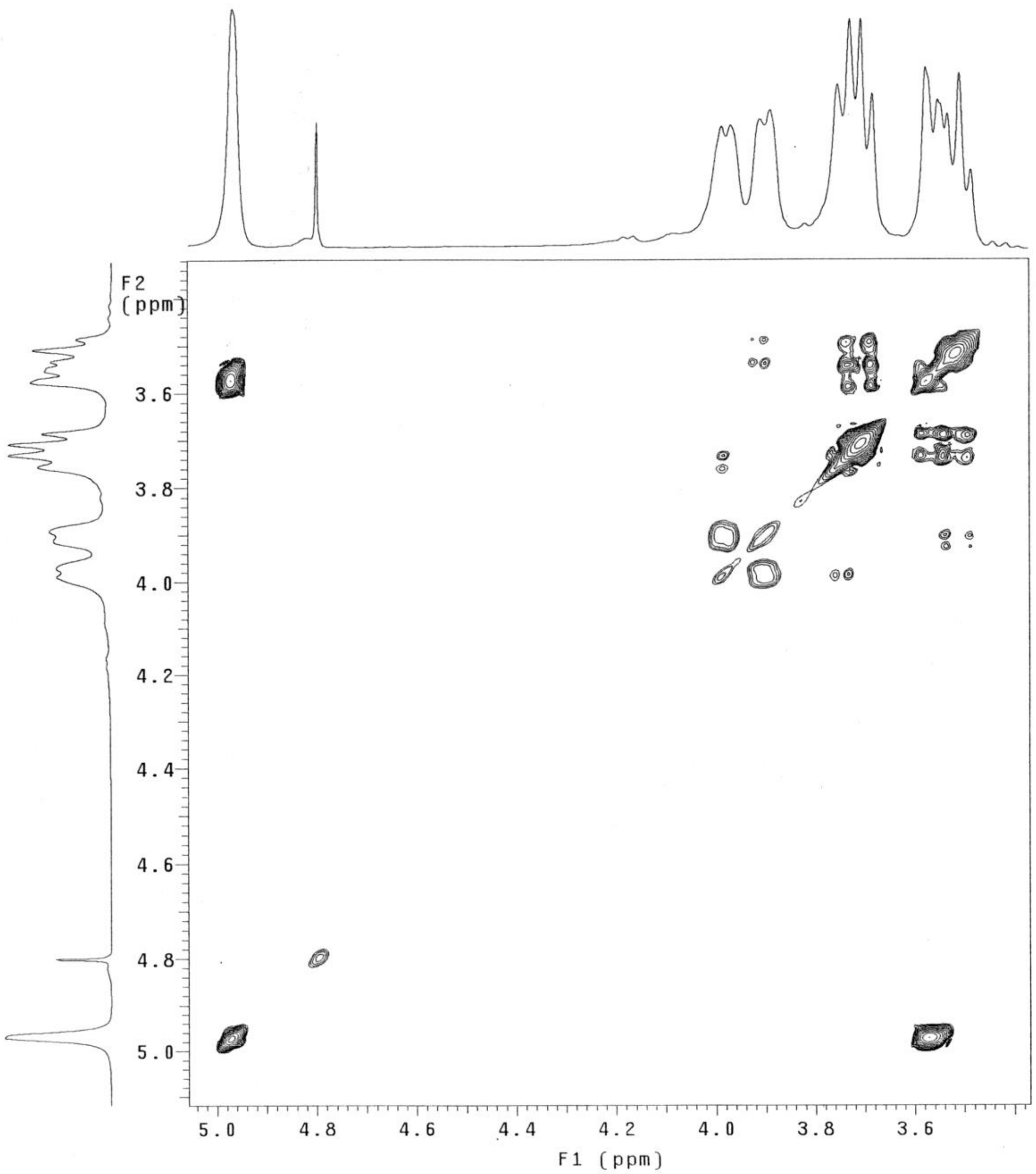

COSY spectrum of EPSB-soluble fraction

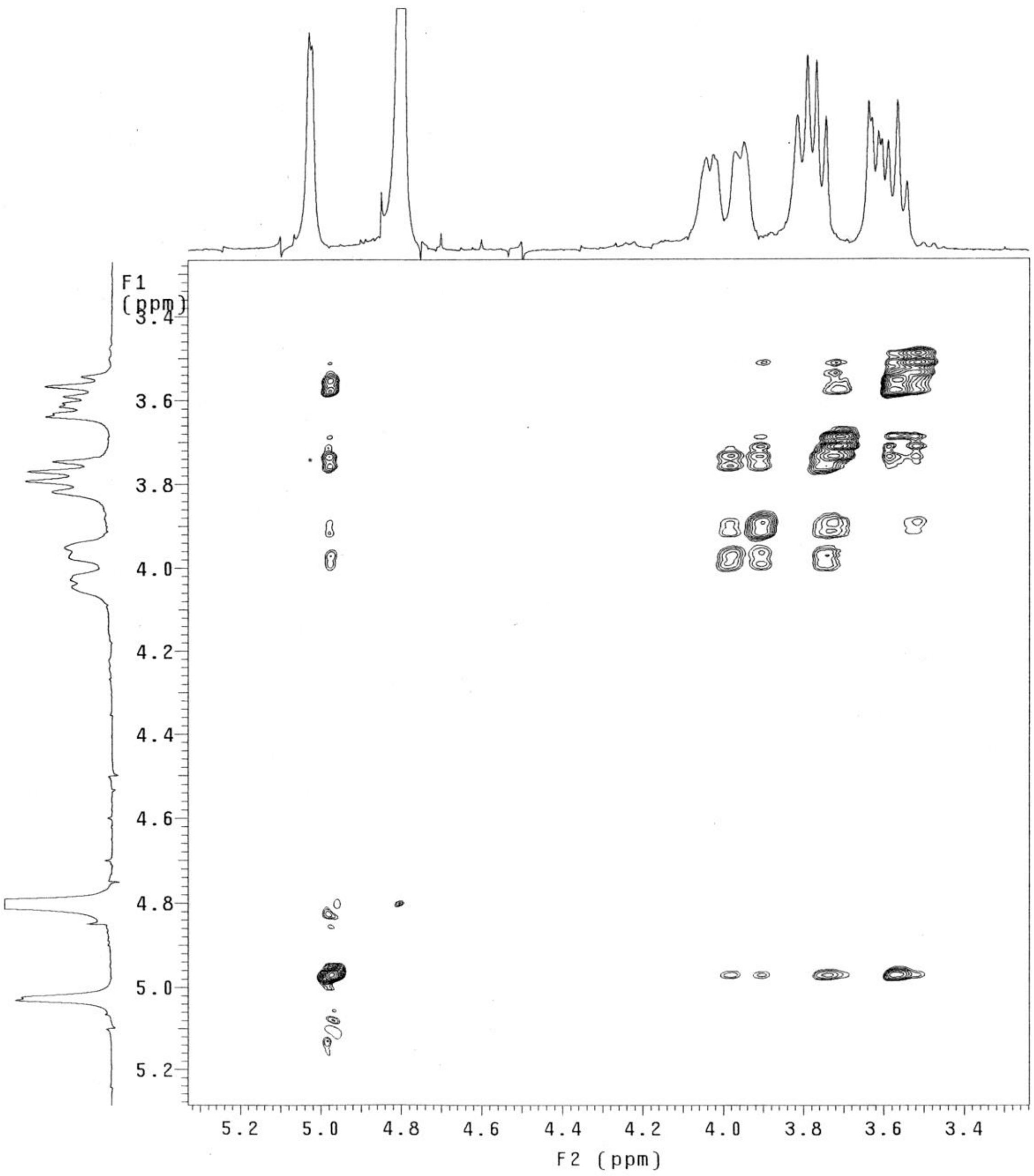

NOESY spectrum of EPSB-soluble fraction

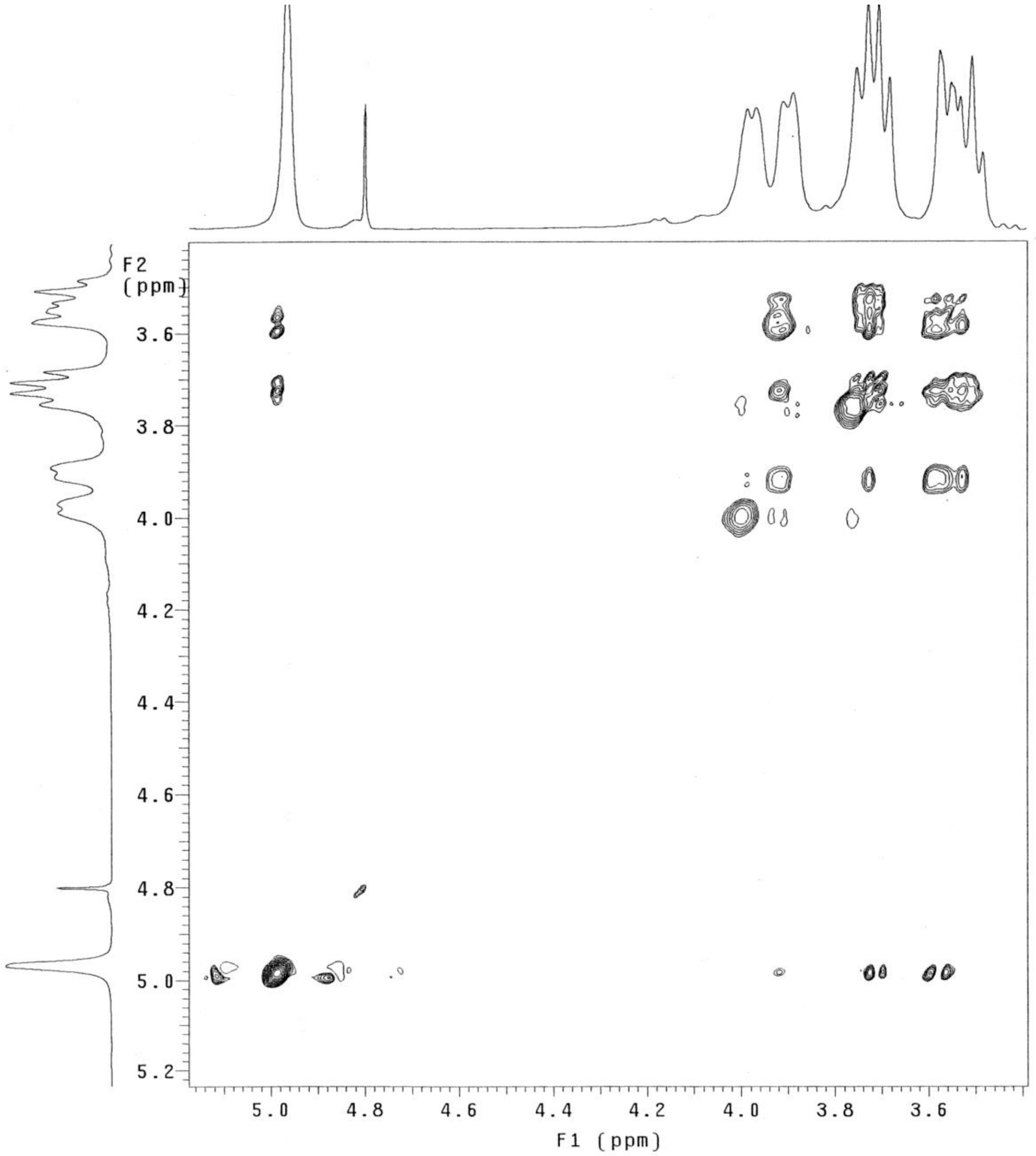

TOCSY spectrum of EPSB-soluble fraction

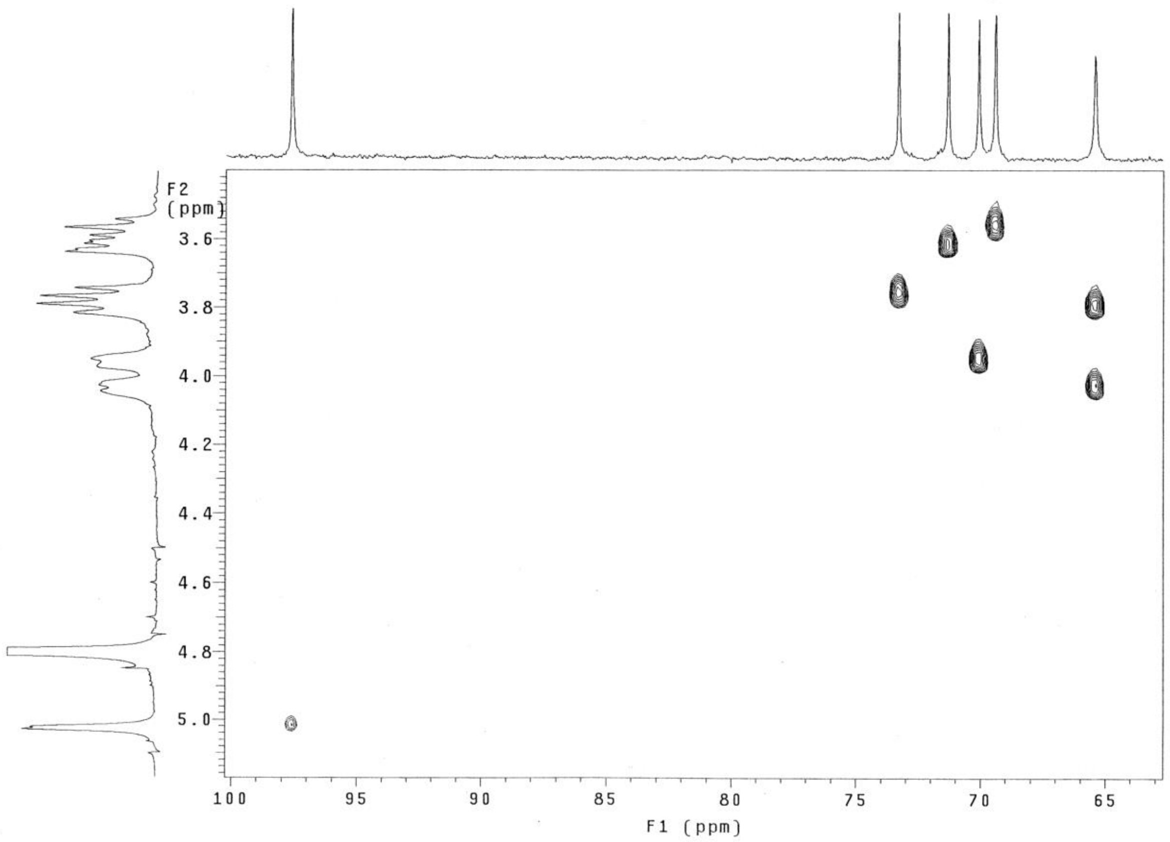

HSQC spectrum of EPSB-soluble fraction

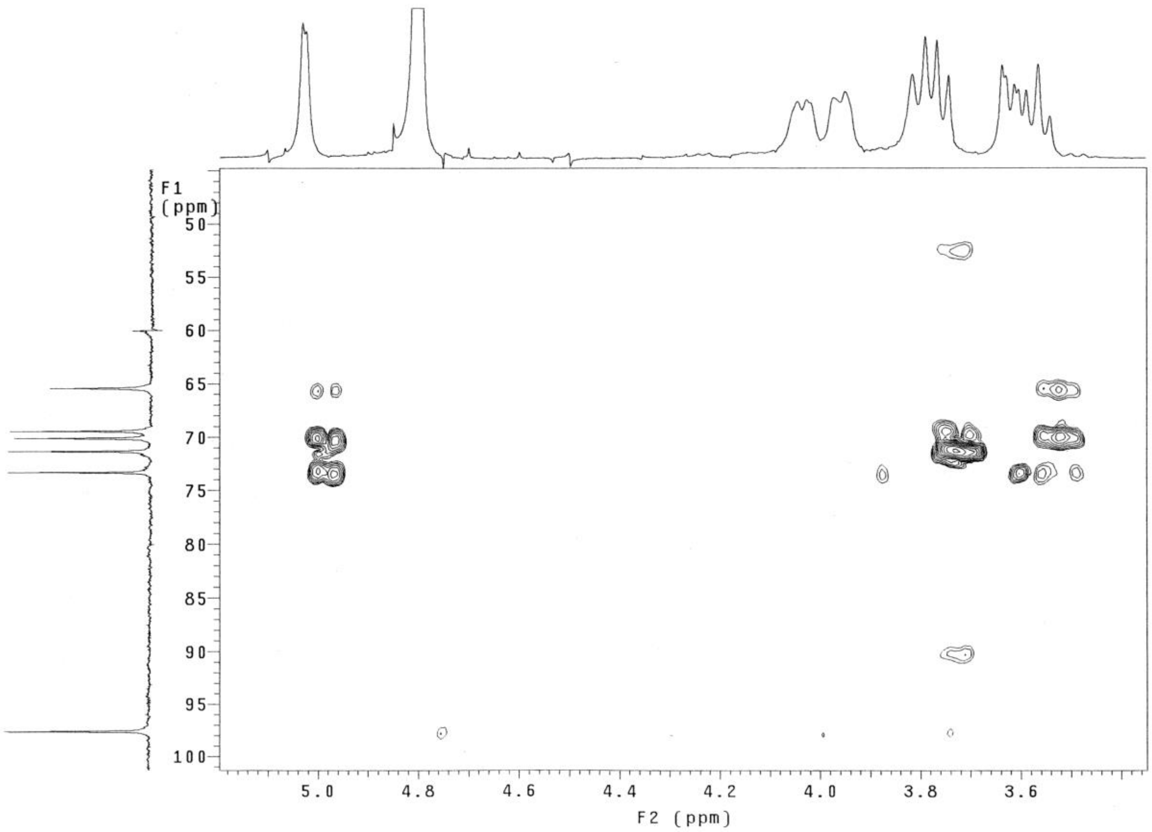

HMBC spectrum of EPSB-soluble fraction

DEPT , ^1H-^1H COSY, NOESY, HSQC and HMBC spectral analysis of EPSB-soluble and cell associated fractions

DEPT spectrum of EPSB-soluble and cell associated fractions

COSY spectrum of EPSB-soluble and cell associated fractions

NOESY spectrum of EPSB-soluble fraction soluble and cell associated fractions

HSQC spectrum of EPSB-soluble and cell associated fractions

HMBC spectrum of EPSB-soluble and cell associated fractions

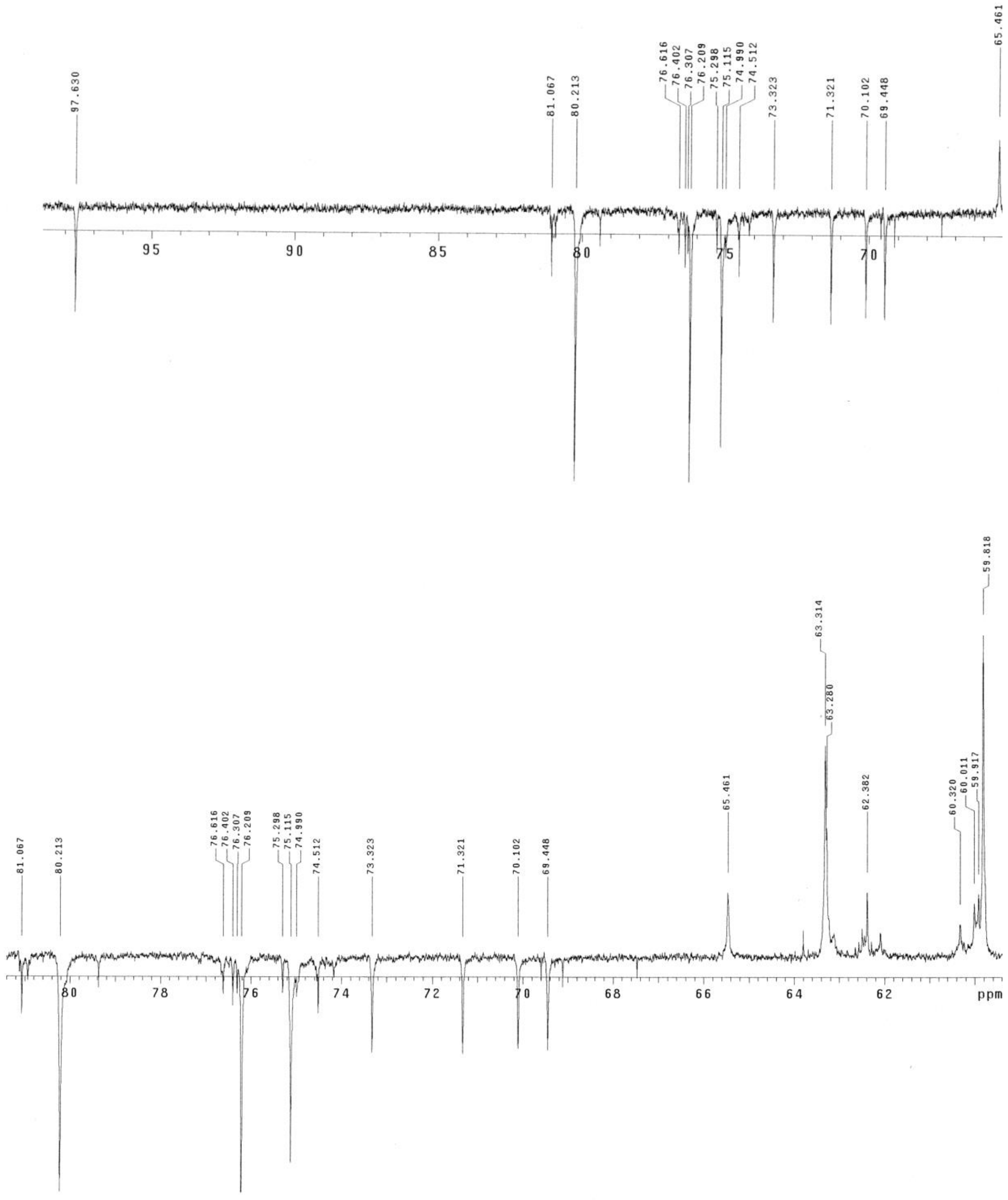

DEPT spectrum of EPSB-soluble and cell associated fractions

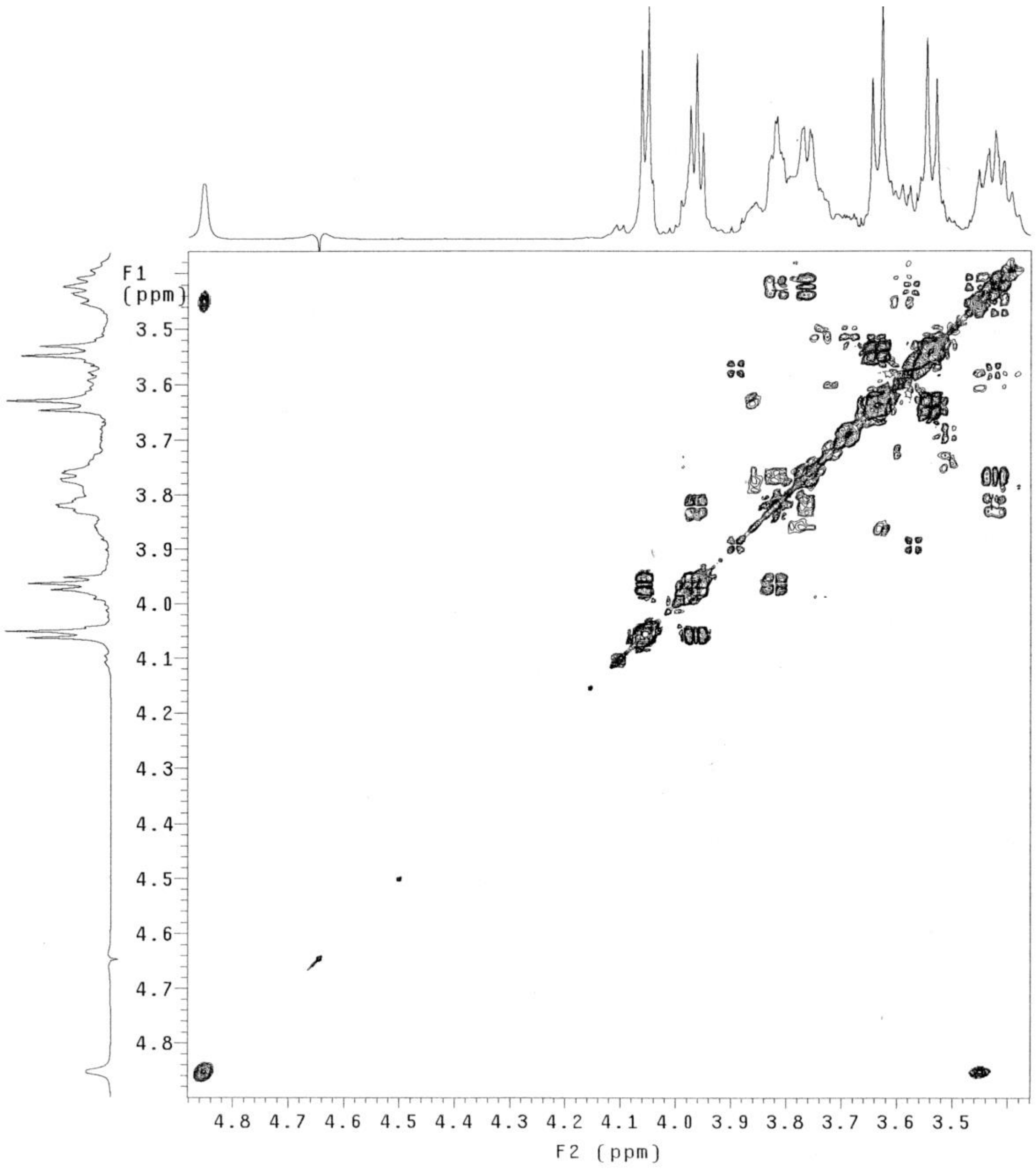

COSY spectrum of EPSB-soluble and cell associated fractions

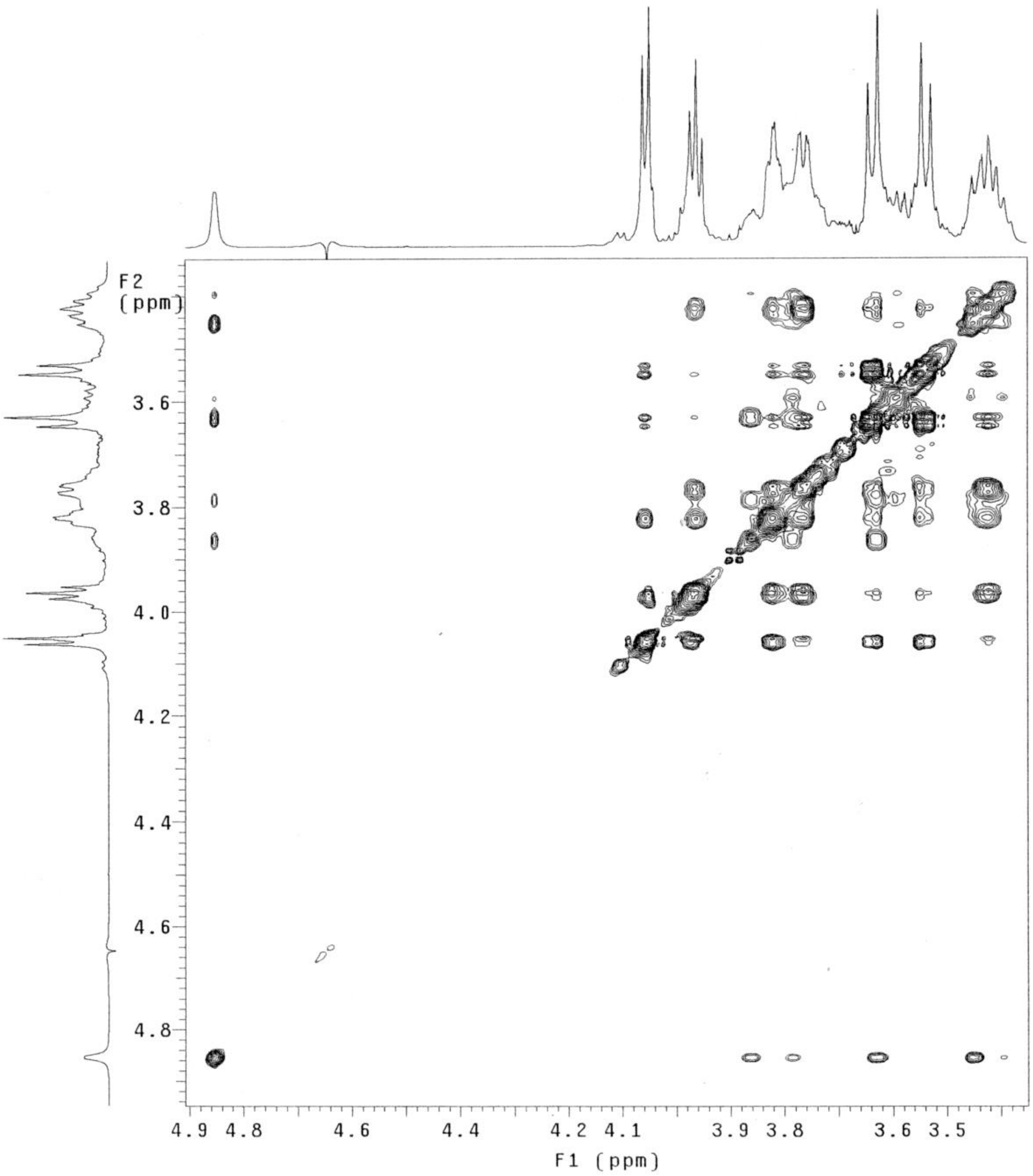

NOESY spectrum of EPSB-soluble and cell associated fractions

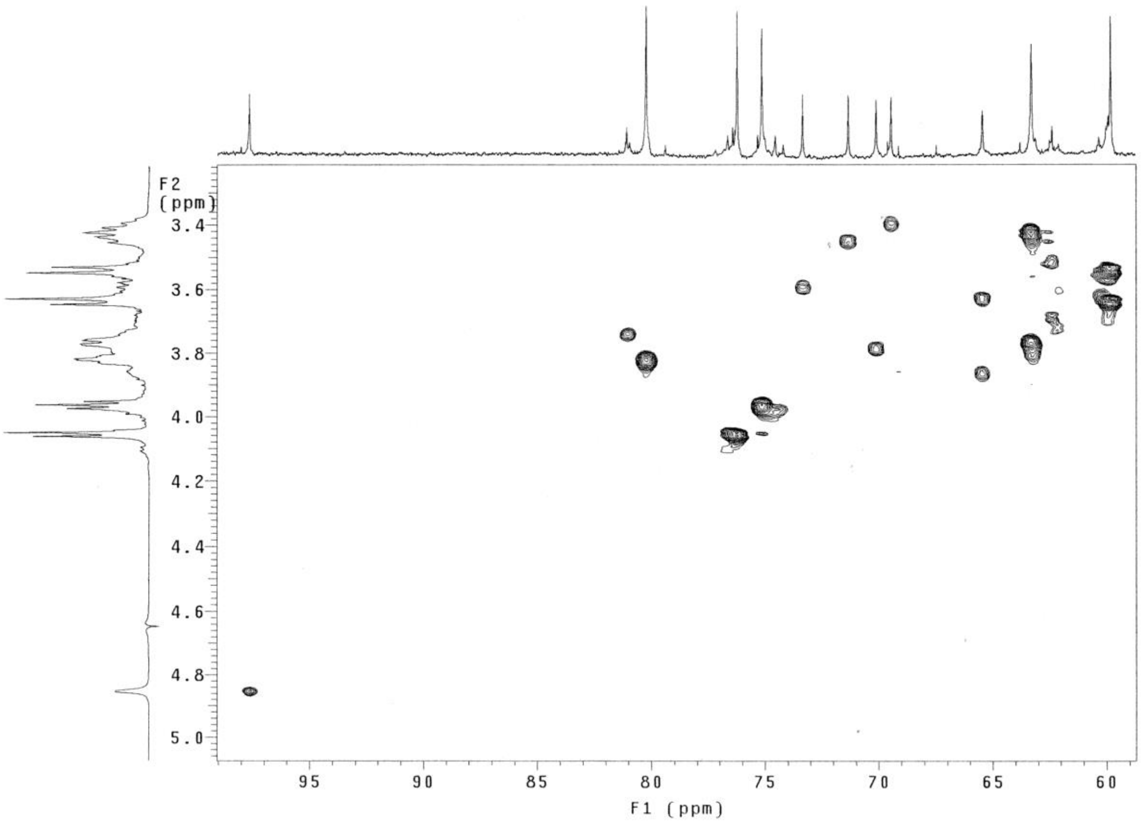

HSQC spectrum of EPSB-soluble and cell associated fractions

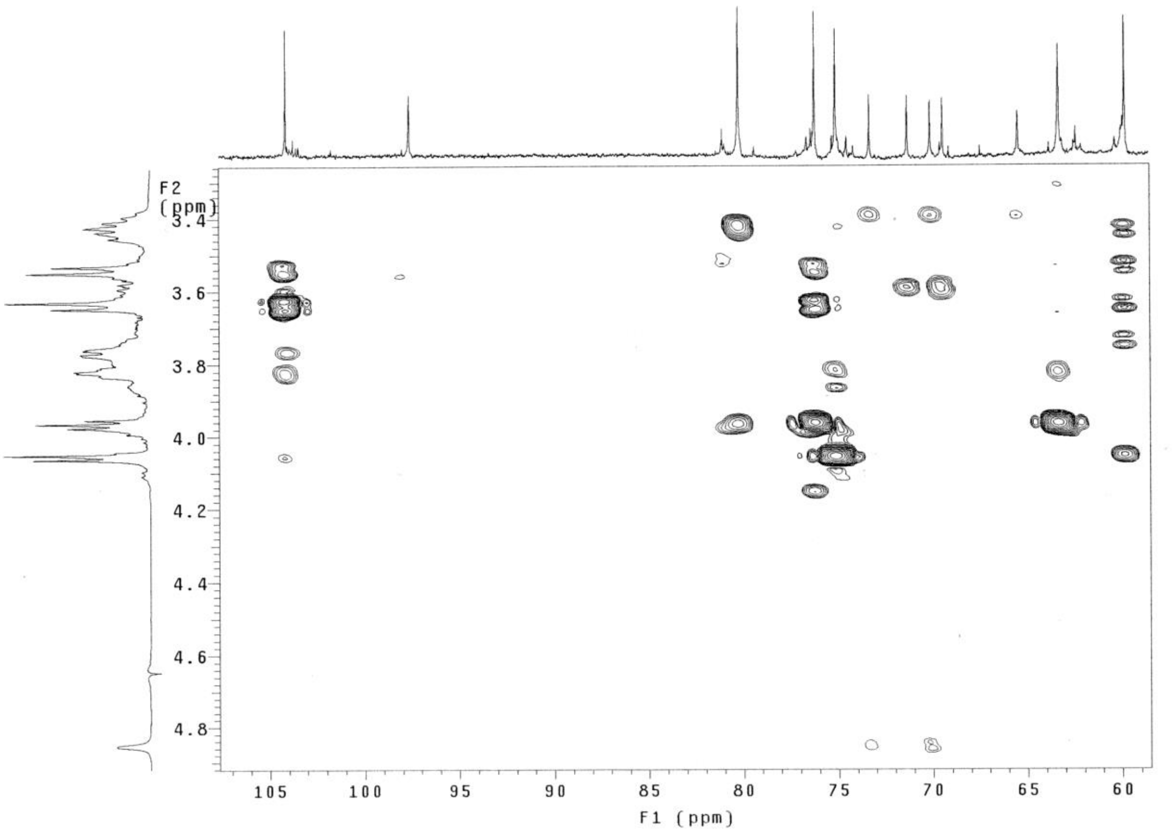

HMBC spectrum of EPSB-soluble and cell associated fractions
